# Formation of cyclic structures in the cationic ring-opening polymerization of 1,3-dioxolane†

**DOI:** 10.1039/d0ra00904k

**Published:** 2020-03-06

**Authors:** Anna M. J. Coenen, Jules A. W. Harings, Samaneh Ghazanfari, Stefan Jockenhoevel, Katrien V. Bernaerts

**Affiliations:** Maastricht University, Faculty of Science and Engineering, Aachen-Maastricht Institute for Biobased Materials (AMIBM), Brightlands Chemelot Campus Urmonderbaan 22 6167 RD Geleen The Netherlands katrien.bernaerts@maastrichtuniversity.nl; RWTH Aachen University, AME-Helmholtz Institute for Biomedical Engineering, Department of Biohybrid & Medical Textiles (BioTex) Forckenbeckstraβe 55 52072 Aachen Germany

## Abstract

The cationic ring-opening polymerization of acetals is prone to cyclization of the polymer chains. This is also the case for the polymerization of 1,3-dioxolane. Literature states that this cyclization can be reduced by applying the Active Monomer mechanism, at least if no competition with the Active Chain End mechanism occurs. In this work, a detailed characterization of the different distributions resulting from the cationic ring-opening polymerization of 1,3-dioxolane *via* the Active Monomer mechanism is made by a combination of gel permeation chromatography, ^1^H NMR, and for the first time by matrix assisted laser desorption/ionization time of flight mass spectrometry. The influence of monomer addition speed, catalyst to initiator ratio and solvent were studied on both kinetics and composition of the product. Furthermore, it was found that increasing the conversion and monomer to initiator ratios leads to an increased amount of cyclic structures and to broader distributions, in correspondence with the Jacobson–Stockmayer theory. Furthermore, ion trapping experiments using ^31^P NMR provide insights into the actual reaction mechanism. Finally, purification of the products after the reactions led to a reduction of the cyclic fraction.

## Abstract 2

Elucidating the mechanism of the cationic ring-opening polymerization of dioxolane using gel permeation chromatography, matrix assisted laser desorption/ionization time of flight mass spectrometry and ^31^P NMR.

## Graphical abstract

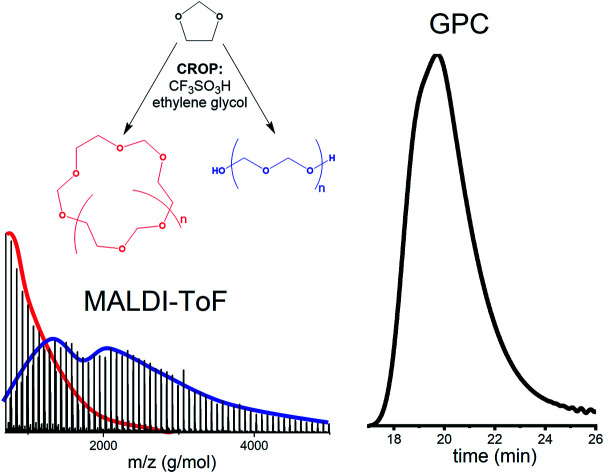

## 1. Introduction

Polydioxolane (PDXL) is a polyether like poly(ethylene glycol) (PEG).^1^ Since PEG is widely used as a material for biomedical applications, PDXL is a potential candidate as well.^2^ Even more, due to the acetal functionalities, PDXL is more prone to acidic degradation, while PEG is not. Therefore, the *in vivo* biodegradation process differs, since this can occur by different mechanisms. However, for many medicinal applications of PDXL further functionalization with, for example, peptides is necessary to improve on either mechanical^3^ or biological functionalities.^3,4^ Since the polymeric backbone of PDXL does not possess reactive groups, only the end-groups of PDXL could be used for functionalization.^5^ Therefore, it is important to be able to make telechelic PDXL, such as α,ω-dihydroxide PDXL.

**Fig. 1:**
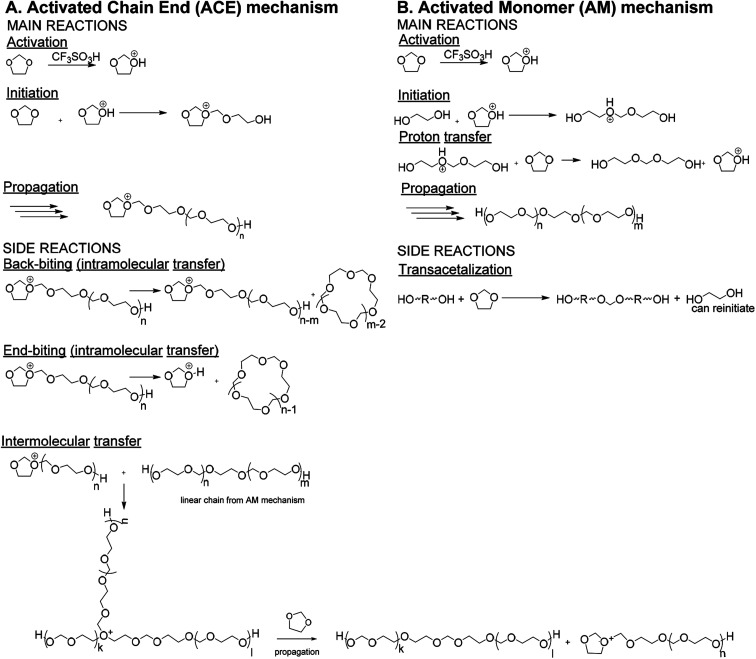
Reaction scheme for CROP of DXL *via* (A) ACE mechanism and (B) AM mechanism.^8^

Traditionally PDXL is synthesized by the cationic ring-opening polymerization (CROP) of DXL *via* the Activated Chain End (ACE) mechanism. However, PDXL chains, as is the case with the polymerization of all cyclic acetals, are prone to cyclization, which broadens the dispersity. If the synthesis of end-functionalized polymers is targeted, the presence of a cyclic fraction that does not contain end-groups is detrimental.^6,7^ The occurrence of cyclics can directly be explained by looking at the mechanism of the DXL polymerization including the side reactions (Fig. 1A). Since the nucleophilicity of the acetal in the monomer is lower compared to that of the acetal functions in the polymer chain, intra- and intermolecular transfer reactions are actually favored over the propagation reaction.^8–10^ This results in large cyclic fractions and broad molecular weight distributions. Jacobson and Stockmayer^11^ developed a theory that allows to predict the molar cyclization equilibrium constant *K*_*x*_ (*x* = ring size) for the ring-chain equilibrium between linear polymer and cyclic oligomers. When the starting concentration of monomers is lower than a critical value (around 0.8 mol L^−1^ (ref. 12)), only cyclic polymers are formed. Above this point, a linear polymer is formed and the proportion of the rings decreases as the concentration increases. Andrews *et al.*^13^ confirmed experimentally that the cycles are oligomeric. The equilibrium concentration of oligomeric cyclics depends on the degree of polymerization of the linear polymer (eqn (1)).^14^

1 [C–M_*x*_] = *K*_*x*_*p*^*x*^

[C–M_*x*_] = concentration of oligomeric cyclics with *x* the number of repeating units, *K*_*x*_ = cyclization equilibrium constant for the formation of *x*-sized rings, *p* = parameter describing the linear polymer namely *p* = 1 − 1/DP_*n*_.

The polymerization of DXL can be controlled by applying the end-blocker method. With this method the chain-transfer reaction is actually used to control the molecular weight of polyacetals.^15^ This method has been applied to obtain telechelic α,ω-bis(methacrylate) PDXL,^6,15^ but cannot be employed to synthesize α,ω-dihydroxide PDXL.

It is reported that cyclization can be prevented when the Active Monomer (AM) mechanism (Fig. 1B)^7,8^ is used, *i.e.* DXL is polymerized by strong protonic acids (*e.g.* triflic acid), in the presence of a diol. The growing chain does not contain a charged active center like in the ACE mechanism, but a hydroxyl group. As the reactivity of the hydroxyl groups towards the active monomer is higher than towards the ether groups of repeating units in the polymer, cyclization reactions are strongly hindered. Since the hydroxyl groups have a higher nucleophilic activity compared to the ether groups in DXL, the diol will initiate the polymerization.^16^ The resulting linear polymer chain contains a protonated ether, which will undergo a proton transfer. This proton transfer occurs fast, therefore reducing the amount of protonated chains drastically compared to the ACE mechanism. Transacetalization side reactions between diols and DXL monomers were shown to occur in the beginning of the reaction.^8,17^ However, the resulting polymer molecules, though doubled in molecular weight, still carry the desired end-standing OH functions.

It has been claimed that proceeding by the AM mechanism would lead to a controlled polymerization in which the total molar mass of the polymer can be defined by the initial diol to monomer ratio.^8,18^ On the other hand, it is well known that the ACE mechanism can occur in parallel with the AM mechanism:^16^ instead of a reaction between diol and activated DXL (AM), an activated DXL unit can also be opened by an uncharged DXL unit (ACE). On top of this, typical side reactions (especially during the ACE polymerization) occur, leading to a mixture of different polymers.

Several authors already described the synthesis of α,ω-dihydroxide PDXL, *e.g.* for the synthesis of crosslinked and non-crosslinked polyurethanes,^8,19,20^ as a precursor for (meth)acrylated bismacromonomers^2,21,22^ or as a macro initiator for the synthesis of triblock copolymers with *e.g.* poly(ethylene oxide).^18^ However, end-group detection was limited to NMR and titration experiments, which do not consider cyclics, and the evolution of the dispersity during the course of the reaction was not studied. It was shown earlier by Jiménez-Pardo *et al.* that for the CROP of trimethylene carbonate *via* the AM mechanism, a combination of gel permeation chromatography (GPC) and matrix-assisted laser desorption/ionization time of flight (MALDI-ToF) was an indispensable tool to elucidate the molecular structure and polymer composition.^23^ Therefore, in this work, a detailed characterization of the different molar mass distributions appearing at several moments of the reaction was done by a combination of GPC and MALDI-ToF measurements. The influence of the reaction parameters on the final distributions in the polymer was also studied.

## 2. Experimental section

### 2.1 Materials

Dichloromethane was obtained from Biosolve and 1,3-dioxolane (99.5%) was obtained from Acros. Both were dried prior to usage by distillation over CaH_2_ (93%, 10–100 mm pieces, up to 10% powder, Acros), and kept on molecular sieves (3 Å, Sigma-Aldrich). Triflic acid (99%) was obtained from Sigma-Aldrich and purified by vacuum distillation (76 °C, 40 mbar) prior to usage. Ethylene glycol (99.8%) was obtained as a dry solvent from Sigma-Aldrich. Triethylamine (>99%), *trans*-2-[3-(4-*tert*-butylphenyl)-2-methyl-2-propenylidene]-malonitrile (>98%), potassium trifluoroacetate (98%), triphenylphosphine (PPh_3_) (99%) and chromium(iii) acetylacetonate (97%) were obtained from Sigma-Aldrich and used as received. The remaining solvents were obtained from Biosolve and used as received.

### 2.2 Polymer synthesis

All polymerizations were performed under N_2_ atmosphere.

#### 2.2.1 Synthesis of PDXL in a thermostated vessel (polymerizations 1–5, 11, 12) (entry 1 Table 1)

Distilled triflic acid was dissolved in dry dichloromethane to obtain a triflic acid solution of 10 μL mL^−1^. The triflic acid solution (1.75 mL/198 μmol) and ethylene glycol (88 μL, 1.57 mmol) were added to dry dichloromethane (3.25 mL) in a thermostated, double-jacketed vessel at 19 °C, to obtain a total amount of 5 mL dichloromethane. Mechanical stirring (150 rpm) was started before adding DXL (143 mmol, 10 mL) to the mixture. Samples of about 1 mL were taken every 5 minutes for the first twenty minutes and then every ten minutes until the end of the reaction. Samples during the reaction were quenched with triethylamine (0.1 mL), to prevent continuation of the reaction; the final sample was taken after quenching the reaction with approximately 2 mL (2.27 mmol) triethylamine. ^1^H-NMR, GPC and MALDI-ToF were measured from the crude samples. Next, the samples were purified by precipitation in diethylether (∼40 mL) and dried to air at ambient temperature. GPC and MALDI-ToF were measured from the purified samples. The product was obtained as a white solid (3.66 g, 35%).

^1^H NMR (300 MHz, CDCl_3_): *δ* 4.77 (2H, –O–C*H*_*2*_–O–), *δ* 3.73 (4H, –O–*CH*_*2*_*–CH*_*2*_–O–, both EG & DXL).

#### 2.2.2 Synthesis of PDXL in a water bath (polymerizations 6–10) (entry 6 Table 1)

Triflic acid (7.5 μL/85 μmol) and ethylene glycol (100 μL, 1.79 mmol) were added to dry dichloromethane (5 mL). The mixture was placed in a water bath at 19 °C and stirring (150 rpm) was started before adding DXL (143 mmol, 10 mL) to the mixture. Samples were taken every hour and quenched with a few drops of triethylamine to prevent continuation of the reaction; the final sample (at 300 minutes) was taken after quenching the reaction with approximately 2 mL (2.27 mmol) triethylamine. ^1^H-NMR and GPC were measured from the crude samples. Next, the samples were purified by precipitation in diethylether (∼40 mL) and dried to air at ambient temperature. GPC and MALDI-ToF were measured from the purified samples. The product was obtained as a white solid (6.57 g, 62%).

^1^H NMR (300 MHz, CDCl_3_): *δ* 4.77 (2H, –O–C*H*_*2*_–O–), *δ* 3.73 (4H, –O–*CH*_*2*_*–CH*_*2*_–O–, both EG & DXL).

### 2.3 Analysis

^1^H-NMR spectra were measured on a Bruker DPX-300 MHz apparatus at ambient probe temperature. Measurements were carried out in CDCl_3_ with 16 scans. Chemical shifts were reported in ppm.

^31^P NMR spectra were measured on a Bruker DPX-300 MHz apparatus at ambient probe temperature. Samples were prepared by mixing a 0.2 mL of a stock solution of 51 mg mL^−1^ PPh_3_ and 34 mg mL^−1^ chromium(iii) acetyl acetonate in DCM with 0.4 mL of unquenched sample from the polymerizations under dry conditions. Spectra were measured using 128 scans and a delay between measurements of 5 s to allow for quantitative measurements. Chemical shifts are reported in ppm.

Gel permeation chromatography (GPC) was measured at 30 °C on a Waters GPC equipped with a Waters 2414 refractive index detector. Tetrahydrofuran was used as eluent at a flow rate of 1 mL min^−1^. Three linear columns were used for separation (Styragel HR1, Styragel HR4 and Styragel HR5, subsequently). The reported molecular weights are relative to poly(ethylene glycol) standards.

Matrix-assisted laser desorption/ionization time-of-flight mass spectrometry (MALDI-ToF MS) was recorded on a Bruker UltrafleXtreme spectrometer with a 355 nm Nd:YAG laser (2 kHz repetition pulse/Smartbeam-II™) and a grounded steel plate. *trans*-2-[3-(4-*tert*-butylphenyl)-2-methyl-2-propenylidene]-malonitrile (20 mg mL^−1^ in THF) and potassium trifluoroacetate (10 mg mL^−1^ in THF) were used as matrix and cation source, respectively. Solid polymers were dissolved in THF (10 mg mL^−1^), for crude mixtures a few drops were dissolved in THF (100 μL). The resulting matrix, salt and polymer solutions were combined in volumetric ratios of 200 : 10 : 30 respectively. All obtained mass spectra were recorded in the reflector mode. The recorded data was processed using the FlexAnalysis (Bruker Daltonics) software package. For calibration an external standard composed of a PEG mixture (*M*_n_ = 1615, 4750 & 10 300 g mol^−1^ (resp. *Đ*: 1.05, 1.11 & 1.04)), α-cyano-4-hydroxycinnamic acid as matrix, and potassium trifluoroacetate as cation source in the same ratios as the samples was used.

## 3. Results & discussion

### 3.1 Limiting conversion

**Fig. 2:**
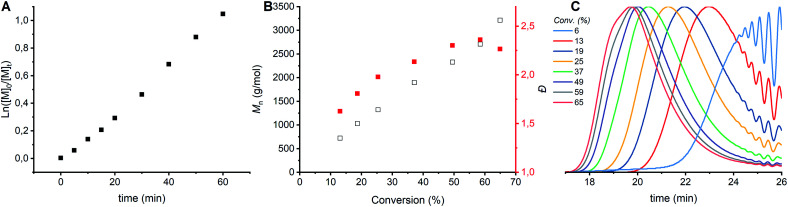
Kinetic study of CROP of DXL with [DXL]_0_ : [ethylene glycol]_0_ : [triflic acid]_0_ = 91 : 1 : 0.13 and [DXL]_0_ = 9.53 mol L^−1^ (entry 1 in Table 1). (A) ln([M]_0_/[M]_*t*_) *vs.* time, (B) *M*_n,GPC_ (black) and dispersity (red) *vs.* % conversion based on ^1^H NMR (C) evolution of normalized GPC traces before precipitation as a function of conversion (THF as eluent, PEG calibration, RI detection).

CROP of cyclic acetals *via* the ACE mechanism is known to lead to a large fraction of cyclics.^6,7,24^ To limit the amount of cyclic structures formed in the polymerizations performed here, ethylene glycol was added as a nucleophile to the protonated monomer in order to let the polymerization proceed by the AM mechanism. The CROP of tetrahydrofuran (THF), a cyclic ether, is prone to cyclization as well, though less than cyclic acetals. In case of THF polymerization, it is known that reducing the conversion to 20% can limit side reactions.^6^ It was hypothesized that limiting the conversion would limit the side reactions in the polymerization of DXL as well. In order to find the optimal conversion, the kinetics of the polymerization were followed by sampling the reaction. ^1^H NMR, GPC and MALDI-ToF analysis were carried out on the crude samples. The first order kinetics (Fig. 2A) and the evolution of *M*_n,GPC_*versus* % conversion (Fig. 2B, black) for entry 1 (Tables 1 and S1 ESI†) shows a linear correlation. Based on only these results no conclusions can be drawn about the occurrence of side reactions. However, the *Đ* increased with increasing conversion and increased molecular weight (Fig. 2B, red). The GPC traces (Fig. 2C) show that the origin of the increase comes from increased lower molecular weight tailing at higher conversion compared to lower conversion. Furthermore, a shoulder at lower elution time appears in the GPC traces at the highest conversions (59 & 65% conversion), which indicates a bimodal distribution.

**Table 1:** Overview of PDXL prepared *via* the AM mechanism with ethylene glycol as initiator at 19 °C

Entry	[DXL]_0_ : [ethylene glycol]_0_ : [triflic acid]_0_	Time (min)	Dropwise monomer addition^a^	[DXL]_0_ (mol L^−1^)	Conv.^c^ (%)	*M*_n,conv._ d (g mol^−1^)	Before precipitation		After precipitation	
							*M*_n,GPC_ e (g mol^−1^)	*Đ* e	*M*_n,GPC_ e (g mol^−1^)	*Đ* e
1	91 : 1 : 0.13	60	No	9.53	65	4450	3200	2.3	4300	1.8
2	181 : 1 : 0.13	60	No	9.53	70	9450	5900	2.4	7700	2.0
3	270 : 1 : 0.13	50	No	9.53	70	14 050	7000	2.7	12 300	1.9
4	358 : 1 : 0.13	50	No	9.53	59	15 700	7000	3.0	13 700	1.9
5	447 : 1 : 0.13	40	No	9.53	48	16 000	4700	4.0	12 600	1.9
6	80 : 1 : 0.05	360	Yes	9.53^b^	89	5350	5200	1.8	5300	1.8
7	80 : 1 : 0.10	180	Yes	9.53^b^	89	5350	4300	1.8	4600	1.7
8	80 : 1 : 0.05	360	No	9.53	83	5000	4000	1.8	4400	1.6
9	80 : 1 : 0.10	180	No	9.53	90	5400	4500	1.8	4800	1.7
10^f^	80 : 1 : 0.10	180	No	14.3	92	5500	4100	2.1	4500	2.0
11	91 : 1 : 0.13	60	No	9.53	51	3500	2400	2.0	—	—
12	91 : 1 : 0.13	60	No	9.53	47	3200	2800	1.7	3700	1.6

a Yes = during 30 minutes.

b Final concentration.

c Conversion was calculated based on ^1^H NMR (ESI eqn (1)).

d
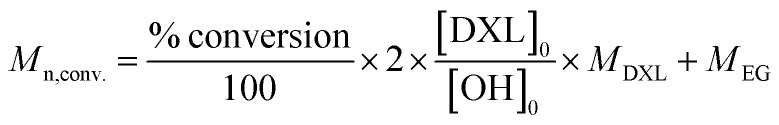
.

e GPC measured in THF (RI detection, PEG calibration).

f Performed without solvent.

**Fig. 3:**
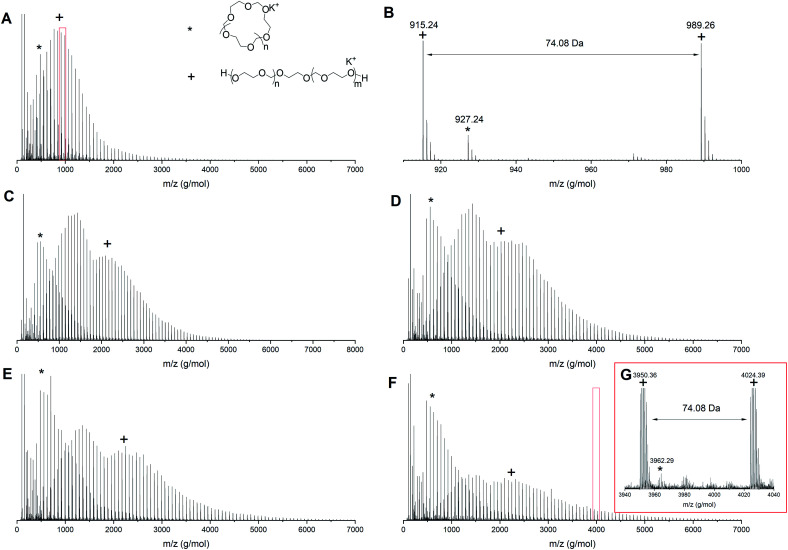
MALDI-ToF spectrum (reflectron mode) for polymerization 1 in Table 1 at different conversions (A) 6% conversion, (B) zoom in of 6% conversion, (C) 19% conversion, (D) 25% conversion, (E) 37% conversion and (F) 65% conversion (G) zoom in of 65% conversion. Linear polymer (C_3_H_6_O_2_)_*n*_C_2_H_6_O_2_K (+); cyclic polymer (C_3_H_6_O_2_)_*n*_K (*).

To understand the origin of the distributions in the multimodal GPC traces better, MALDI-TOF analysis was performed on the intermediate samples. Fig. 3 shows the MALDI-ToF spectra for PDXL prepared *via* the AM mechanism (entry 1, Table 1) at different conversions. At 6% conversion (Fig. 3A and zoom in Fig. 3B) two distributions can be seen, one at high molecular weight, which can be assigned to the desired linear PDXL with two hydroxyl end groups and one at low molecular weight, which can be assigned to the cyclic byproduct and corresponds to the low molecular weight tailing in GPC. Though AM polymerization should not result in cyclics, the ACE mechanism that competes with the AM mechanism accounts for the formation of cyclic structures.^16^

As the reaction proceeds cyclics of a molecular weight up to 4000 g mol^−1^ can be observed (Fig. 3G). At the same time the distribution assigned to the linear PDXL shows bimodal behavior (Fig. 3C–F) and broadening of the dispersity, as was also observed in GPC. The high molecular weight part of the bimodal distribution also corresponds to the desired OH functionalized telechelic PDXL. The distribution most probably originates from further propagation reactions on the tertiary oxonium ions formed upon intermolecular transfer reactions^25^ happening during the ACE mechanism that competes with the AM mechanism (Fig. 1).

This increase in cyclic formation with increasing conversion as visualized in both GPC and MALDI-ToF is in correspondence with the findings of Jacobson and Stockmayer.^11^ The polymerization should ideally be terminated before any transfer reactions are occurring to obtain the most controlled polymerization, even though cyclization is happening throughout the reaction. For polymerization 1, this means the reaction should be terminated between 6 and 19% conversion.

### 3.2 Formation of cyclic structures *versus* different targeted molecular weights

**Fig. 4:**
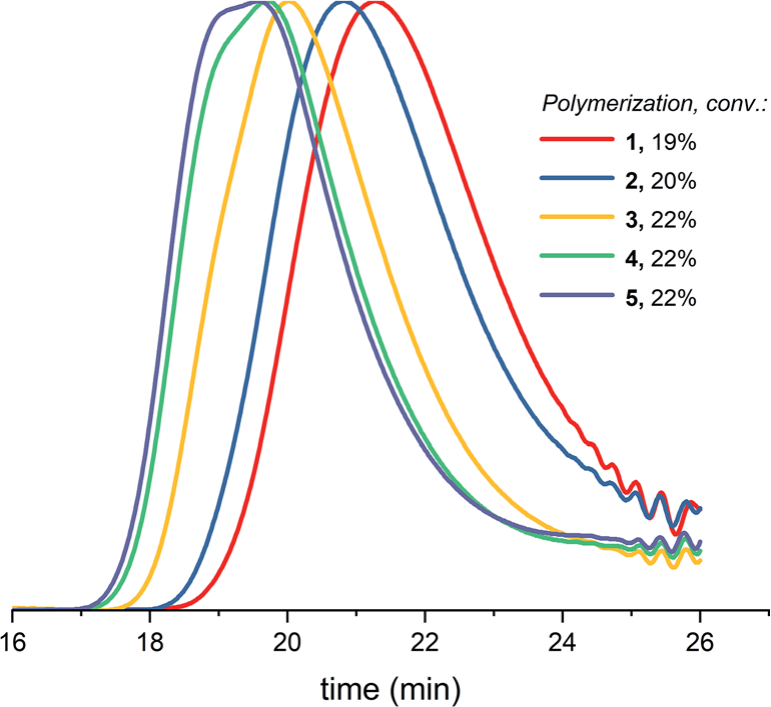
Normalized GPC traces (THF as eluent, PEG calibration, RI detection) for DXL polymerizations with increasing targeted molecular weights (1–5 in Table 1) at similar conversion before precipitation.

Table 1 (entry 1–5) reveals that the difference between *M*_n,conv._ and *M*_n,GPC_ (before precipitation) as well as the dispersity increase upon increasing the targeted molecular weight. The GPC traces of 5 polymerizations with different targeted molecular weights at similar conversion (Fig. 4) demonstrate increased low molecular weight tailing, broadening of the curve and eventually a bimodal distribution when increasing the targeted molecular weight. Just as with increased conversion, as showed in paragraph 3.1, the cyclization side reactions increase upon increasing the targeted molecular weight, in correspondence with eqn (1) of the Jacobson–Stockmayer theory and with the findings of Penczek *et al.*^26^ Penczek *et al.*^26^ state that at the lower concentration of the alcohol initiator required to achieve high theoretical degrees of polymerization, the contribution of the AM mechanism will be decreased at the expense of increased contribution of the ACE mechanism including the corresponding side reactions.

### 3.3 Influence of monomer addition speed and catalyst to initiator ratio

Since limiting the conversion could not completely prevent the formation of cyclic structures in the polymerization of DXL, the influence of different reaction conditions on the formation of cyclics was investigated.

It is reported that the formation of cyclics and thus the final composition of the obtained polymers is dependent on the instantaneous concentration of the non-protonated monomer.^27,28^ As mentioned earlier, there is a competition between the AM and the ACE mechanism. The AM mechanism is favored in monomer-starved conditions, since most of the monomer will then be in its protonated form and cannot act as a nucleophile. To influence the [DXL]/[DXL]^+^ ratio, two different parameters were investigated. First, the monomer addition speed was varied. Slow addition (over 30 min) would according to literature lead to a lower [DXL]/[DXL]^+^ ratio favoring the AM mechanism. Second, the catalyst to initiator ratio was adjusted, more catalyst would increase [DXL]^+^ thus lowering the ratio as well.

**Fig. 5:**
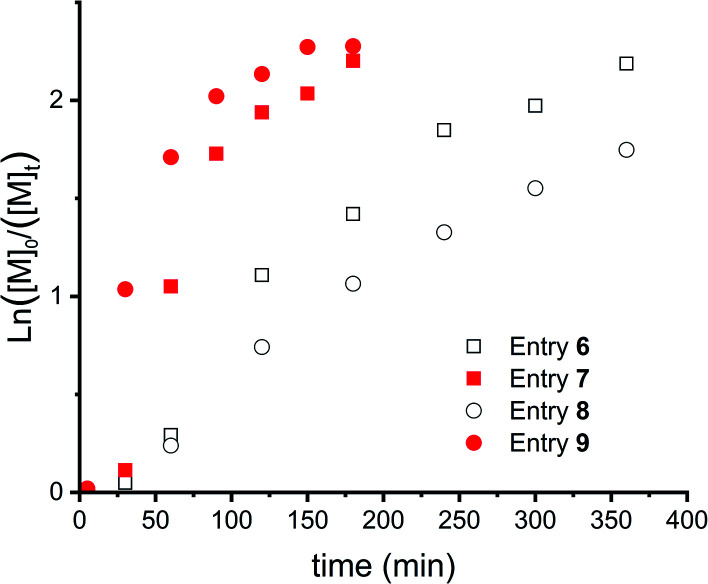
First order kinetic plots for CROP of DXL (entries 6–9 in Table 1). Squares for slow DXL addition (over 30 min) and circles for DXL addition at once. Open symbols for an initiator : catalyst = 1 : 0.05 and closed symbols for initiator : catalyst = 1 : 0.10.

Fig. 5 shows the kinetic study of 4 polymerizations where these parameters were varied. First, slow monomer addition (over 30 minutes, entries 6 and 7, squares) is compared with fast addition (below 1 minute, entries 8 and 9, circles). This showed a delayed onset, as can be seen for both polymerizations, this delay is longer for slow addition of the monomer (entries 6 and 7). However, this difference can most likely be explained by the lower monomer concentration in the beginning of the reaction for slow addition. Furthermore, a lower catalyst to initiator ratio decreased the reaction speed and resulted in more controlled polymerizations, as concluded from the more linear evolution of the first order kinetics (Fig. 5).

**Fig. 6:**
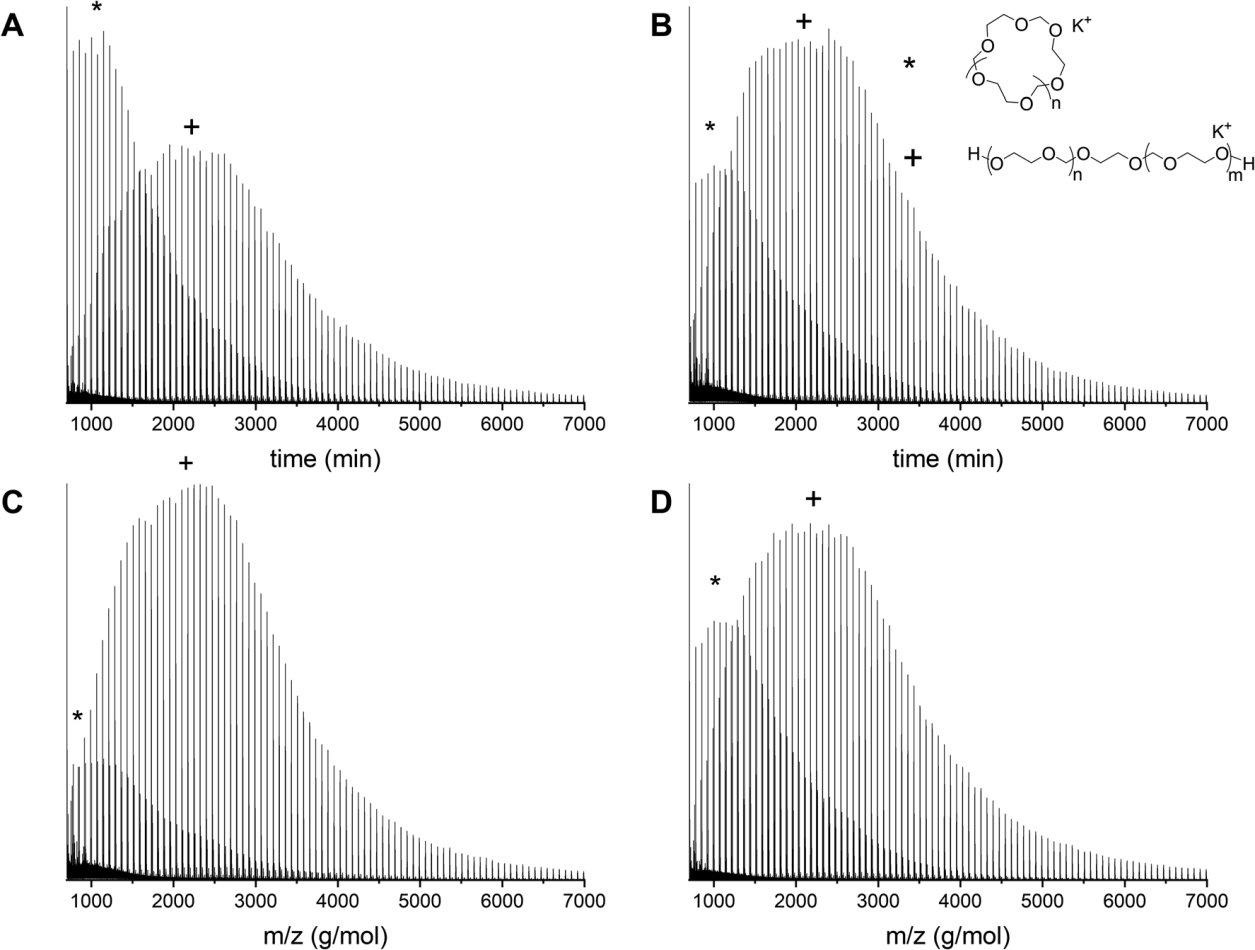
Overview of the MALDI-ToF spectra (reflectron mode) of PDXL with (A) [DXL]_0_ : [ethylene glycol]_0_ : [triflic acid]_0_ = 80 : 1 : 0.05 and slow monomer addition (entry 6 in Table 1), (B) [DXL]_0_ : [ethylene glycol]_0_ : [triflic acid]_0_ = 80 : 1 : 0.10 and slow monomer addition (entry 7 in Table 1), (C) [DXL]_0_ : [ethylene glycol]_0_ : [triflic acid]_0_ = 80 : 1 : 0.05 and fast monomer addition (entry 8 in Table 1) and (D) [DXL]_0_ : [ethylene glycol]_0_ : [triflic acid]_0_ = 80 : 1 : 0.10 and fast monomer addition (entry 9 in Table 1). Linear polymer (C_3_H_6_O_2_)_*n*_C_2_H_6_O_2_K (+); cyclic polymer (C_3_H_6_O_2_)_*n*_K (*).

GPC showed large *Đ* for all these polymerizations (Tables 1 and S2, ESI†) and MALDI-ToF analysis confirmed that none of these circumstances were sufficient to avoid the cyclization during the polymerization since both cyclics and linear structures could be identified in all reactions (Fig. 6). As can be seen, neither the monomer addition speed, nor the catalyst to initiator ratio influenced the formation of cyclics in a significant way. This is in correspondence with a recent MALDI-ToF study on the ring-opening polymerization of cyclic carbonates, which showed no difference between multi-feed step and single-feed step addition of the monomer.^23^

### 3.4 Bulk polymerization

**Fig. 7:**
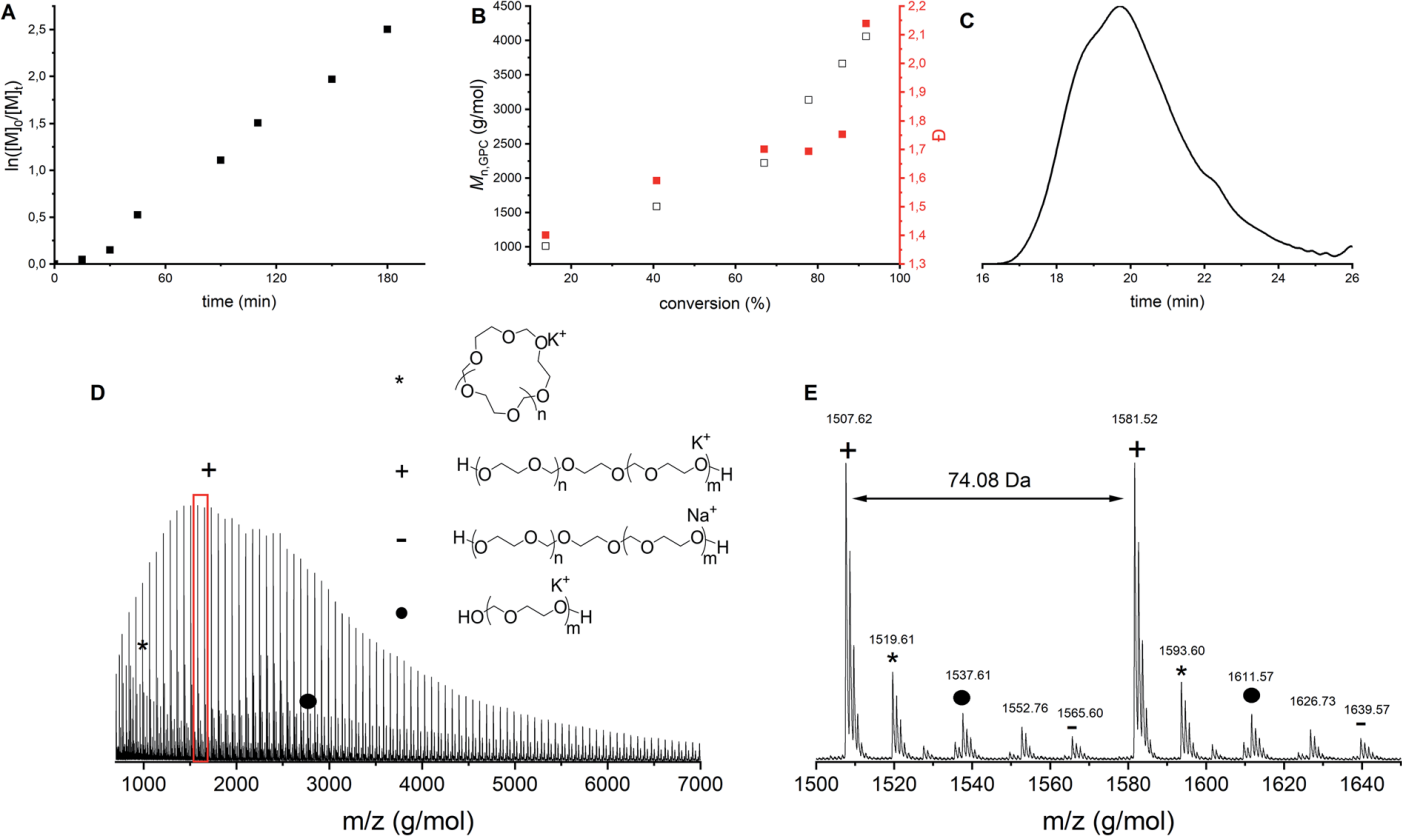
CROP of DXL in bulk with [DXL]_0_ : [ethylene glycol]_0_ : [triflic acid]_0_ = 80 : 1 : 0.1 and [DXL]_0_ = 14.3 mol L^−1^ (entry 10 in Table 1). (A) ln([M]_0_/[M]_*t*_) *vs.* time, (B) *M*_n,GPC_ (black) and dispersity (red) *vs.* % conversion based on ^1^H NMR, (C) typical GPC trace (THF as eluent, PEG calibration, RI detection) before precipitation, (D) MALDI-ToF spectrum (overview), (E) MALDI-ToF spectrum (detailed) (measured in reflectron mode) with linear polymer (C_3_H_6_O_2_)_*n*_C_2_H_6_O_2_K (+) and (C_3_H_6_O_2_)_*n*_C_2_H_6_O_2_Na (−); cyclic polymer (C_3_H_6_O_2_)_*n*_K (*); linear polymer *via* ACE (C_3_H_6_O_2_)_*n*_H_2_OK (•).

Franta *et al.* reported that at a monomer concentration below 0.85 mol L^−1^, only cyclics are formed due to the thermodynamic control on the polymerization reaction.^8^ Following this line of thought, it was hypothesized that a higher monomer concentration could lead to less cyclics. To investigate this claim, polymerization 10 was carried out without solvent, increasing the monomer concentration to 14.3 mol L^−1^ (Table 1). Although the first order kinetic plot (Fig. 7A) and the molecular weight evolution as function of conversion (Fig. 7B, black) are rather linear, the dispersities increase (Fig. 7B, red). This indicates that more and more side reactions are happening as a function of conversion, as also confirmed by the shape of the GPC trace (Fig. 7C). MALDI-ToF analysis (Fig. 7D and E) showed indeed multiple distributions. First, both the linear and cyclic polymers that were identified in all previous polymerizations were found as well (indicated by star and plus signs). However, in this bulk polymerization more distributions are seen than during solution polymerization (Fig. 3). Not all distributions could be assigned, but one of them corresponds to the polymer synthesized by the ACE mechanism (Fig. 7D and E, dot). This product was present not only at the end of the reaction, but could already be identified at low conversions.

### 3.5 Ion-trapping experiments

The previously described reactions show the formation of cyclics, but direct evidence for either the AM or ACE mechanism cannot be provided. Fig. 1 shows that when reactions proceed *via* the ACE mechanism mostly tertiary oxonium ions are present, whereas with the AM mechanism only secondary oxonium ions can be found. Brzezinska *et al.* showed that these ions can be identified using ^31^P NMR after trapping the ions using PPh_3_.^29^ Polymerization 11 (Table 1 and ESI Table S3†) was carried out to identify the presence of secondary and or tertiary oxonium ions. A reaction between a tertiary oxonium ion and PPh_3_ results in RP^+^Ph_3_CF_3_SO_3_^−^ with R being a polymer chain. When PPh_3_ reacts with a secondary oxonium ion HP^+^Ph_3_CF_3_SO_3_^−^ is formed instead. However, it was noticed that no differentiation could be made between the unreacted PPh_3_ compound and the protonated variant, due to fast proton exchange between the two compounds. Still the chemical shift at which the combined peak appears is related to the ratio between the two compounds.^30^ Therefore both the chemical shift of the PPh_3_/HP^+^Ph_3_CF_3_SO_3_^−^ peak and the ratio between the two integrals for tertiary (around −4 ppm) and quaternary phosphonium salts (around 18.5 ppm) can be used for a qualitative analysis of the presence of rep. secondary and tertiary oxonium ions, even though no quantitative analysis can be made.

**Fig. 8:**
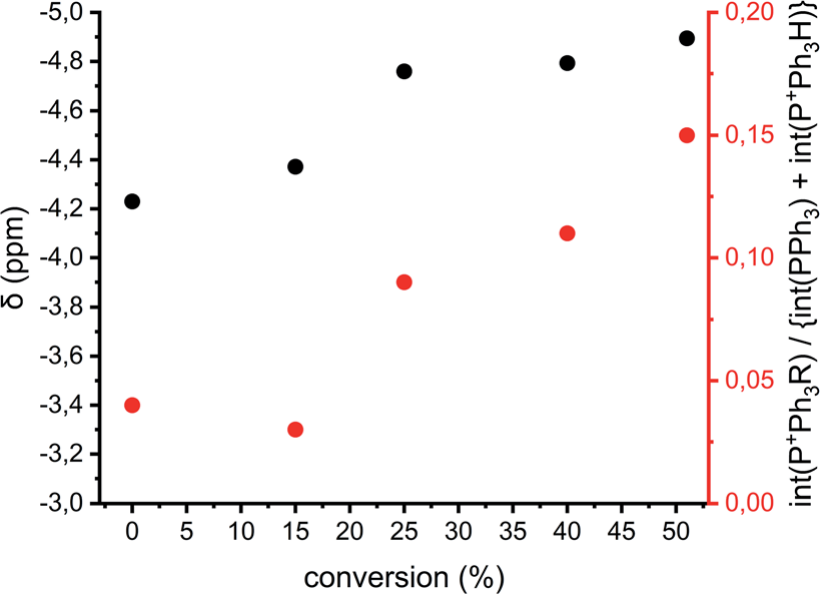
Analysis of the ^31^P NMR data of polymerization 11 as a function of conversion. Black: chemical shifts of the PPh_3_/HP^+^Ph_3_CF_3_SO_3_^−^ peak. Red: the integral of RP^+^Ph_3_CF_3_SO_3_^−^ peak with the integral PPh_3_/HP^+^Ph_3_CF_3_SO_3_^−^ peak set as 1.

Fig. 8 shows the chemical shifts of the PPh_3_/HP^+^Ph_3_CF_3_SO_3_^−^ peak and the relative integrals of quaternary *vs.* tertiary (and PPh_3_) phosphonium ions of polymerization 11 as a function of conversion (based on the spectra in the ESI Fig. S16†). It can be seen that the PPh_3_/HP^+^Ph_3_CF_3_SO_3_^−^ peak shifts upfield during the polymerization, this implies that the amount of HP^+^Ph_3_CF_3_SO_3_^−^ decreases during the reaction. By consequence the amount of secondary oxonium ions decrease indicating less prevalence of the AM mechanism. This goes hand in hand with an increase in the peak intensity of RP^+^Ph_3_CF_3_SO_3_^−^ indicating an increase in tertiary oxonium ions. This suggests that the occurrence of the ACE mechanism increases when the polymerization progresses and thus the occurrence of side reactions will increase as well.

### 3.6 Purification of polymerization mixtures

**Fig. 9:**
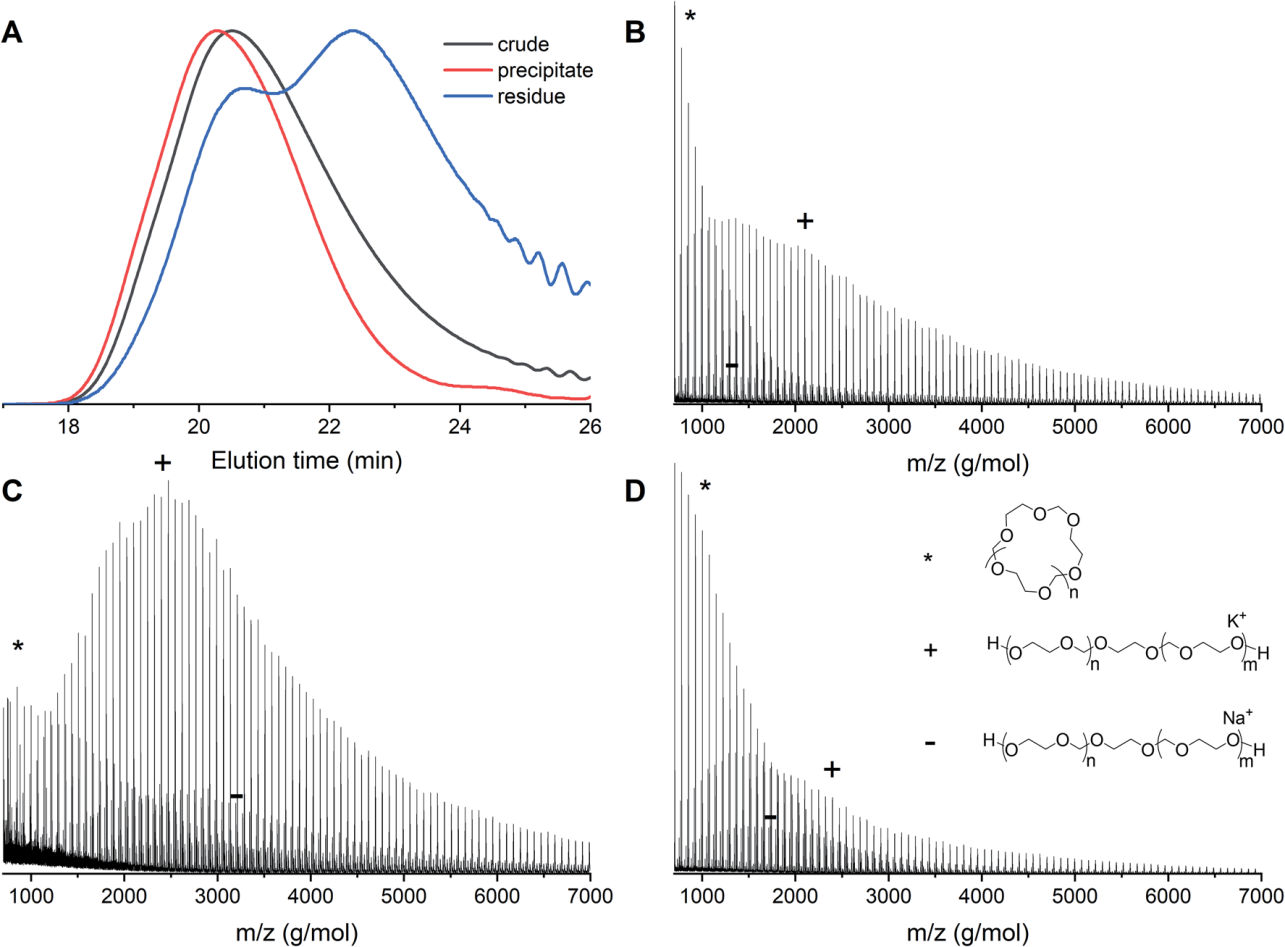
Entry 12 (Table 1) with [DXL]_0_ : [ethylene glycol]_0_ : [triflic acid]_0_ = 91 : 1 : 0.13 at 48% conversion before and after precipitation. (A) Normalized GPC traces (THF as eluent, PEG calibration, RI detection) (B) MALDI-ToF spectrum of crude before precipitation (C) MALDI-ToF spectrum of precipitate (D) MALDI-ToF spectrum of residue (reflectron mode). Linear polymer (C_3_H_6_O_2_)_*n*_C_2_H_6_O_2_K (+) and (C_3_H_6_O_2_)_*n*_C_2_H_6_O_2_Na (−); cyclic polymer (C_3_H_6_O_2_)_*n*_K (*).

In order to remove the cyclic fraction from the polymers, the reaction mixtures were purified by precipitation in diethyl ether. This leads to a suspension of polymer in the solution and a fraction of powder that sinks immediately to the bottom of the vial. The suspension was discarded and the residual material was analyzed by both MALDI-ToF and GPC. Precipitation leads to higher molecular weights and decreased dispersities as a consequence of the removal of cyclic PDXL. The GPC traces before and after precipitation of polymerization 12 show that the tailing disappears and the peak becomes more narrow (Fig. 9A). This is confirmed by Table 1, which shows decreased dispersity and increased molecular weight upon precipitation. It can indeed be seen in Fig. 9D that the residue contains mainly lower molecular weight polymers (linear and cyclic).

The GPC results are confirmed by MALDI-ToF spectra. The MALDI-ToF spectrum before purification (Fig. 9B) shows clearly distributions of cyclic polymers at the lower molecular weight (star) and linear polymers at the higher molecular weight (plus and minus). The MALDI-ToF spectrum after precipitation (Fig. 9C) shows a significantly larger quantity of linear polymers when compared to the cyclic polymers. In addition, in Fig. 9D, it can be seen that mostly lower molecular weight polymers and a large cyclic fraction are present in the residue. This indicates that part of the cyclic fraction has been removed from the polymer mixture. It can also be seen that a part of the linear fraction at low molecular weight is discarded as well. The yield of precipitation was approximately 70%, however, based on the GPC and MALDI-ToF data, most of the discarded polymers would have been the undesired cyclic structures.

When samples of higher molecular weight were purified using precipitation, this effect was not as significant as for the samples with lower molecular weight. In these cases, the precipitation did not lead to a suspension, but to a clear solution, in combination with powder at the bottom of the vial. These heavier polymers precipitated faster and are therefore more likely to lead to entrapment of cyclics in the obtained powder. Still, the MALDI-ToF spectra of these higher molecular weight samples before and after purification showed reduced cyclics (ESI, Fig. S6–S15†). However, the dispersity measured with GPC was quite large, eventhough it had become significantly smaller upon precipitation (ESI Table S1 and Fig. S3, S5†). To obtain highly purified samples for these higher molecular weights, more precipitations steps will be necessary.

## 4. Conclusion

The formation of cyclic structures as a side product during cationic ring-opening polymerization of DXL, limits the preparation of telechelic diol functionalized PDXL, whose end groups are often necessary for further functionalization. To study the formation of these cyclic PDXL structures, MALDI-ToF MS was utilized for the first time in combination with GPC and ^1^H NMR. MALDI-ToF MS proved to be an ideal method to study the composition of the polymerization products, which led to a better insight in the polymerization of DXL *via* the Active Monomer (AM) mechanism.

Analyzing the polymerization of DXL during the reaction with both MALDI-ToF and GPC showed an increase in *Đ* upon increase of both conversion and targeted molecular weight. At low molecular weight side, cyclic PDLX is formed due to the competition between the AM mechanism and the ACE mechanism. At high molecular weight side bimodality in the GPC and MALDI spectra can be explained by the presence of the desired diol functionalized PDXL as well as diol functionalized PDXL originating from intermolecular transfer reactions happening during the ACE mechanism.

Furthermore, this combination of analytical techniques showed that changing the reaction conditions (monomer addition speed, catalyst to initiator ratio, and solvent) did not prevent cyclization from occurring during the polymerization of DXL *via* the AM mechanism. In the end, the best results were obtained at low catalyst to initiator ratio's, by working in solvent and limiting the conversion.

Even though the use of the AM mechanism compared to ACE polymerization should reduce the formation of cyclics, it can be concluded that cyclization still occurs when utilizing the AM to synthesize PDXL, even when slow monomer addition is applied what should normally suppress ACE. Ion trapping experiments using ^31^P NMR show that the ACE mechanism becomes more prevalent with increasing conversion, which implies that more side reactions are taking place with increasing conversion as well. Precipitation into diethyl ether allows purification of the polymer by reducing the cyclic fraction.

## Conflicts of interest

There are no conflicts to declare.

## Supplementary Material

RA-010-D0RA00904K-s001
