# Supplementary material for: Formation of cyclic structures in the cationic ring-opening polymerization of 1,3-dioxolane
Source: RSC Adv. 2020 Mar 6;10(16):9623–32. doi: 10.1039/d0ra00904k (PMC9050153; doi:10.1039/d0ra00904k)
Supplement: RA-010-D0RA00904K-s001 [file RA-010-D0RA00904K-s001.pdf]

Electronic Supplementary Material (ESI) for RSC Advances.
This journal is © The Royal Society of Chemistry 2020

Supporting Information

Formation of cyclic structures in the cationic ring opening polymerization of 1,3-dioxolane

Anna M. J. Coenen, Jules A. W. Harings, Samaneh Ghazanfari, Stefan Jockenhoevel, Katrien V. Bernaerts

Table of contents:

NMR analysis.. 2

GPC data versus % conversion 3

GPC traces of crude samples of polymerizations **2-11**.................... 6

GPC traces of purified samples of polymerizations **1-5**.................. 8

MALDI-ToF MS of crude samples of polymerizations **2-5**, **11** measured in reflectron mode.......... 9

MALDI-ToF MS of purified samples of polymerizations **1-5,** measured in reflectron mode............ 14

^{31}P spectra of polymerization **11** 17

^{1}H NMR analysis

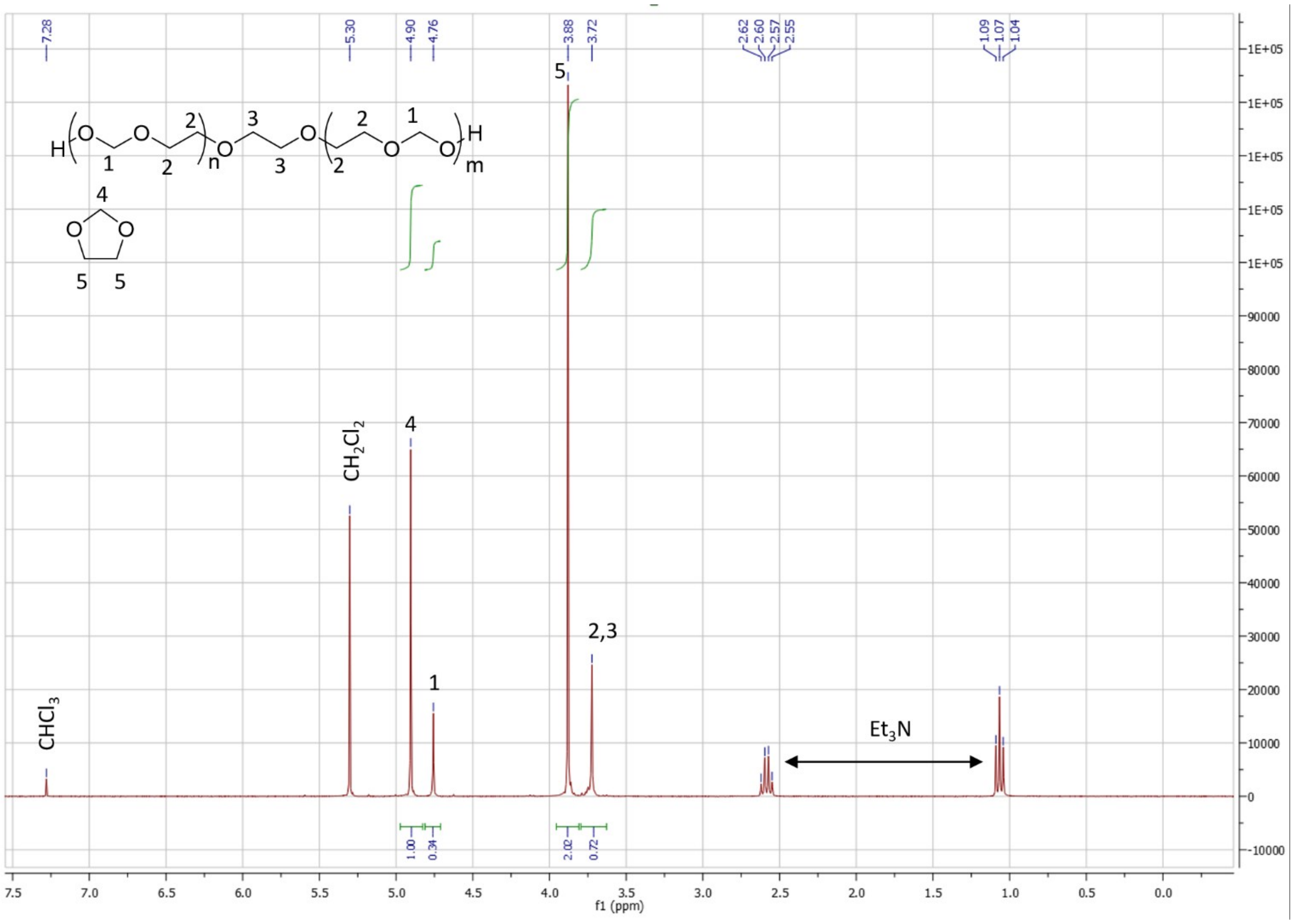

Figure S1. Typical ^{1}H NMR spectrum to calculate conversion for the CROP of dioxolane.

$$\% \text{ conversion} = \frac{1H_{polymer}}{1H_{monomer} + 1H_{polymer}} * 100 = \frac{2H_1/2\ (\delta = 4.75\ ppm)}{2H_4/2\ (\delta = 4.90\ ppm)\ +\ 2H_1/2\ (\delta = 4.75\ ppm)} * 100 \quad (1)$$

GPC data versus % conversion

Table S1. Overview of the GPC results before and after precipitation for different reactions linked to conversion and $M_{n,conv}$: polymerizations 1-5.

Polymerization	Time (min)	Conversion[a] (%)	$M_{n,conv}$ [b] (g/mol)	Before precipitation $M_{n,GPC}$ (g/mol)	Before precipitation *Đ*	After precipitation $M_{n,GPC}$ (g/mol)	After precipitation *Đ*
**1**	8	6	450	-	-	-	-
	15	13	950	700	1.6	-	-
	23	19	1350	1000	1.8	-	-
	30	25	1750	1300	2.0	3200	1.3
	38	37	2550	1900	2.1	3600	1.4
	45	49	3350	2300	2.3	3900	1.6
	53	59	4000	2700	2.4	4000	1.8
	60	65	4400	3200	2.3	4300	1.8
**2**	8	5	750	-	-	-	-
	15	10	1400	900	1.9	-	-
	23	20	2750	1800	1.9	-	-
	30	26	3550	2400	2.1	4200	1.5
	38	39	5250	4100	2.0	5200	1.7
	45	52	7000	5200	2.1	5800	2.0
	53	62	8350	5900	2.2	6800	2.0
	60	70	9400	5900	2.4	7800	2.0
**3**	8	7	1450	700	1.8	-	-
	15	13	2650	1700	1.8	-	-
	23	22	4450	2500	2.2	4900	1.4
	30	30	6050	3200	2.5	6200	1.6
	38	46	9300	5600	2.6	8100	1.8
	45	61	12300	5800	2.7	10000	1.9
	53	70	14100	7000	2.7	12300	1.9
**4**	8	4	1100	700	1.4	-	-
	15	9	2450	1600	1.6	-	-
	23	15	4050	1800	2.4	-	-
	30	22	5900	2700	2.6	5800	1.5
	38	35	9300	4300	2.9	8500	1.7
	45	51	13550	5600	3.1	11300	1.8
	53	59	15650	7000	3.0	13740	1.9
**5**	8	4	1400	700	1.7	-	-
	15	8	2700	1000	2.3	-	-
	23	15	5000	2200	2.3	4700	1.4
	30	22	7350	2200	3.4	6200	1.5
	38	35	11650	3500	6.6	9200	1.8
	45	48	16000	4700	4.2	12600	1.9

[a] Conversion was calculated based on ^{1}H NMR (Supplementary information equation 1)

[b] $$M_{n,conv} = \frac{\%\ conversion}{100} * \frac{[DXL]_0}{[initiator]_0} * M_{DXL} + M_{initiator}$$

Table S2. Overview of the GPC results before precipitation for different reactions linked to conversion and $M_{n,conv}$: polymerizations 6-10.

				Before precipitation	
**Polymerization**	**Time (min)**	**Conversion[a] (%)**	**$M_{n,conv}$ [b] (g/mol)**	**$M_{n,GPC}$ (g/mol)**	**$Đ$**
**6**	30	5	350	-	-
	60	25	1550	1600	1.4
	120	67	4050	3800	1.8
	180	76	4550	4500	1.8
	240	84	5050	4500	1.9
	300	86	5200	4700	1.9
	360	89	5300	5200	1.8
**7**	30	11	700	-	-
	60	65	3950	3300	1.6
	90	82	4950	3900	1.8
	120	86	5150	4100	1.8
	150	87	5250	4100	1.8
	180	89	5350	4300	1.8
**8**	5	1	100	-	-
	60	21	1350	1200	1.4
	120	52	3200	2100	1.9
	180	66	3950	2600	2.1
	240	73	4450	3600	1.8
	300	79	4750	3800	1.8
	360	83	5000	4000	1.8
**9**	5	2	200	-	-
	30	65	3900	3100	1.7
	60	82	4950	4000	1.8
	90	87	5200	4200	1.8
	120	88	5300	4100	1.9
	150	90	5400	4100	1.8
	180	90	5400	4500	1.8
**10**	15	5	350	-	-
	30	14	900	1000	1.4
	45	41	2500	1600	1.6
	90	67	4050	2200	1.7
	110	78	4700	3200	1.7
	150	86	5200	3700	1.8
	180	92	5500	4100	2.1

[a] Conversion was calculated based on ^1H NMR (Supplementary information equation 1)

[b] $$M_{n,conv} = \frac{\%\, conversion}{100} * \frac{[DXL]_0}{[initiator]_0} * M_{DXL} + M_{initiator}$$

Table S3. Overview of the GPC results before precipitation linked to conversion and $M_{n,conv}$ for polymerization 11.

Polymerization	Time (min)	$[DXL]_0$ (mol/L)	Conversion[a] (%)	$M_{n,conv}$ [b] (g/mol)	Before precipitation $M_{n,GPC}$[c] (g/mol)	$Đ$
**11**	0	9.53	0	0	-	-
	20	9.53	15	1100	800	1.6
	30	9.53	25	1700	1300	1.7
	45	9.53	40	2800	1900	1.9
	360	9.53	51	3500	2400	2.0

[a] Conversion was calculated based on ^{1}H NMR (Supplementary information equation 1)

[b] $$M_{n,conv} = \frac{\%\,conversion}{100} * \frac{[DXL]_0}{[initiator]_0} * M_{DXL} + M_{initiator}$$

[c] GPC traces are shown in Figure S4.

GPC traces of crude samples of polymerizations 2-5 & 6-10

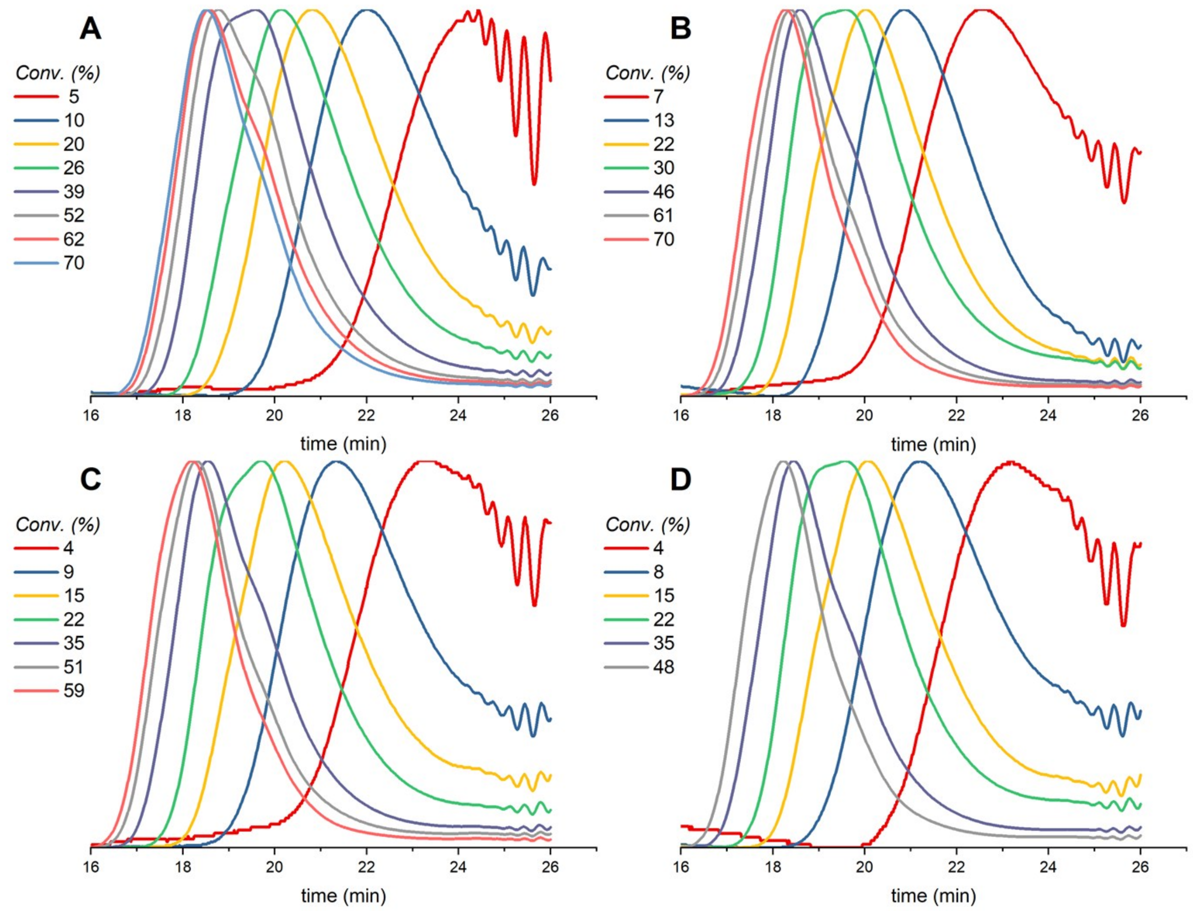

Figure S2. GPC traces (THF as eluent, PEG calibration, RI detection) of crude samples taken during polymerizations (A) 2, (B) 3, (C) 4, (D) 5 (Table 1).

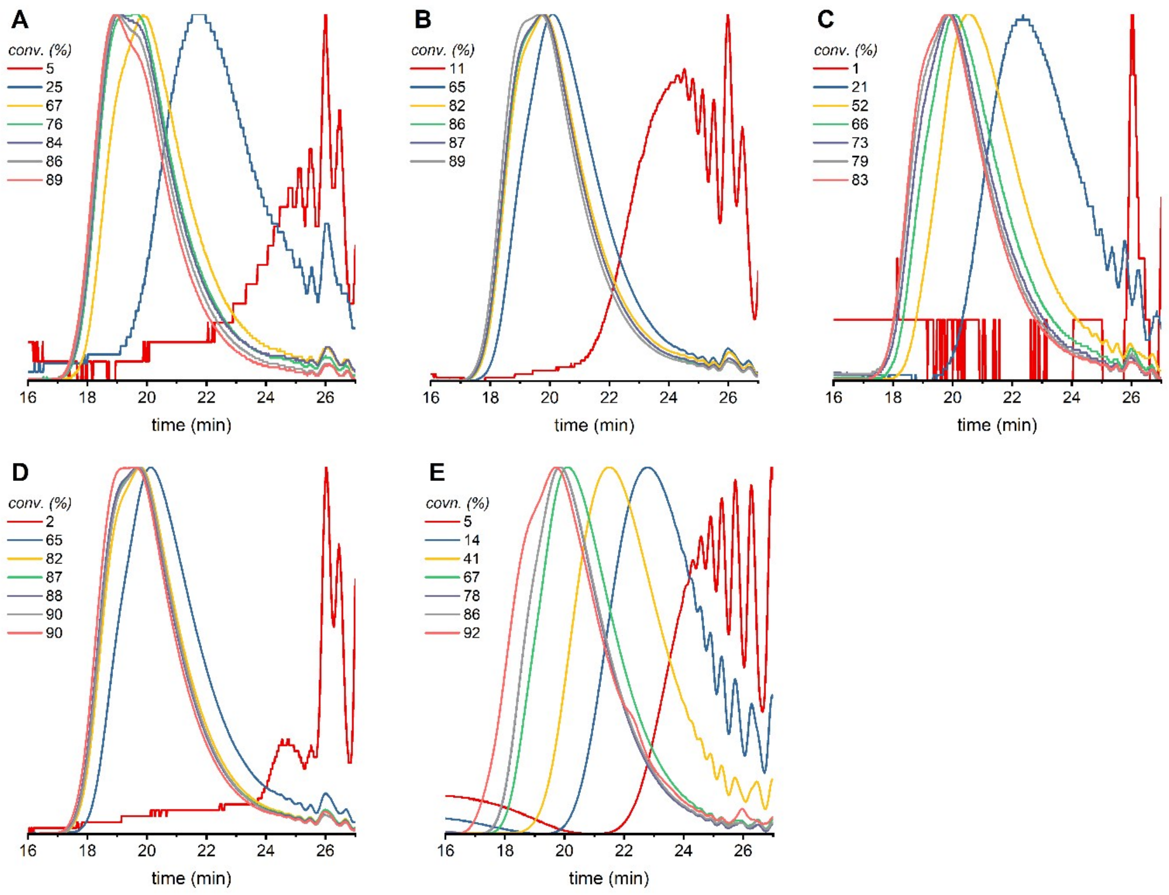

Figure S3 GPC traces (THF as eluent, PEG calibration, RI detection) of crude samples taken during polymerizations (A) 6, (B) 7, (C) 8, (D) 9, (E) 10 (Table 1).

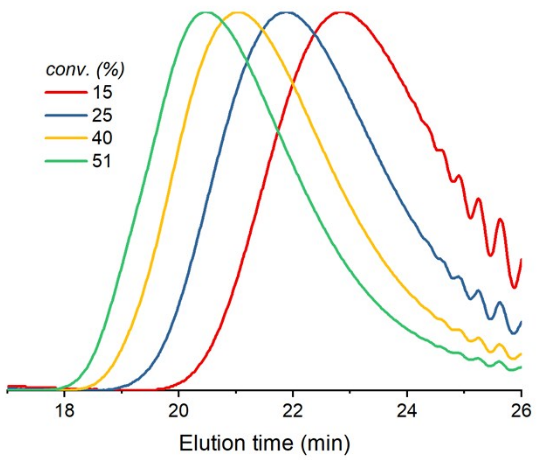

Figure S4 GPC traces (THF as eluent, PEG calibration, RI detection) of crude samples taken during polymerization 11

GPC traces of purified samples of polymerizations 1-5

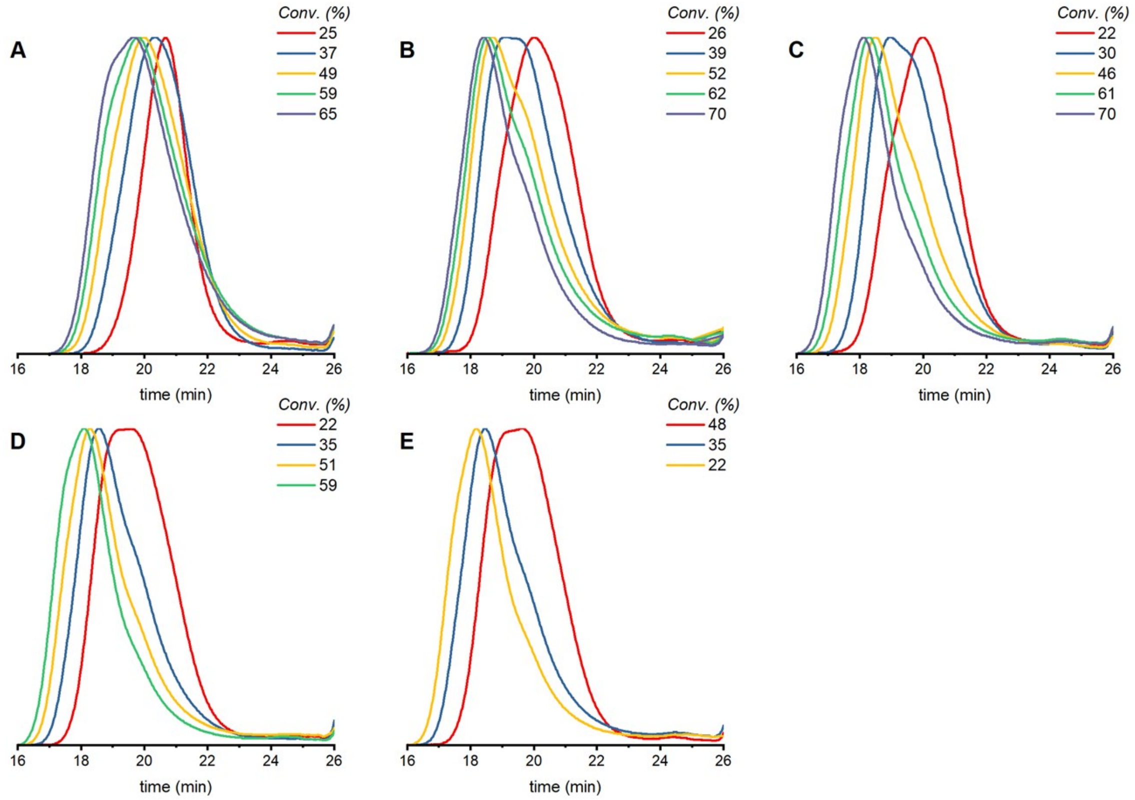

Figure S5 GPC traces (THF as eluent, PEG calibration, RI detection) of purified samples taken during polymerization (A) 1, (B) 2, (C) 3, (D) 4, (E) 5 (Table 1).

MALDI-ToF MS of crude samples of polymerizations 2-5, measured in reflectron mode

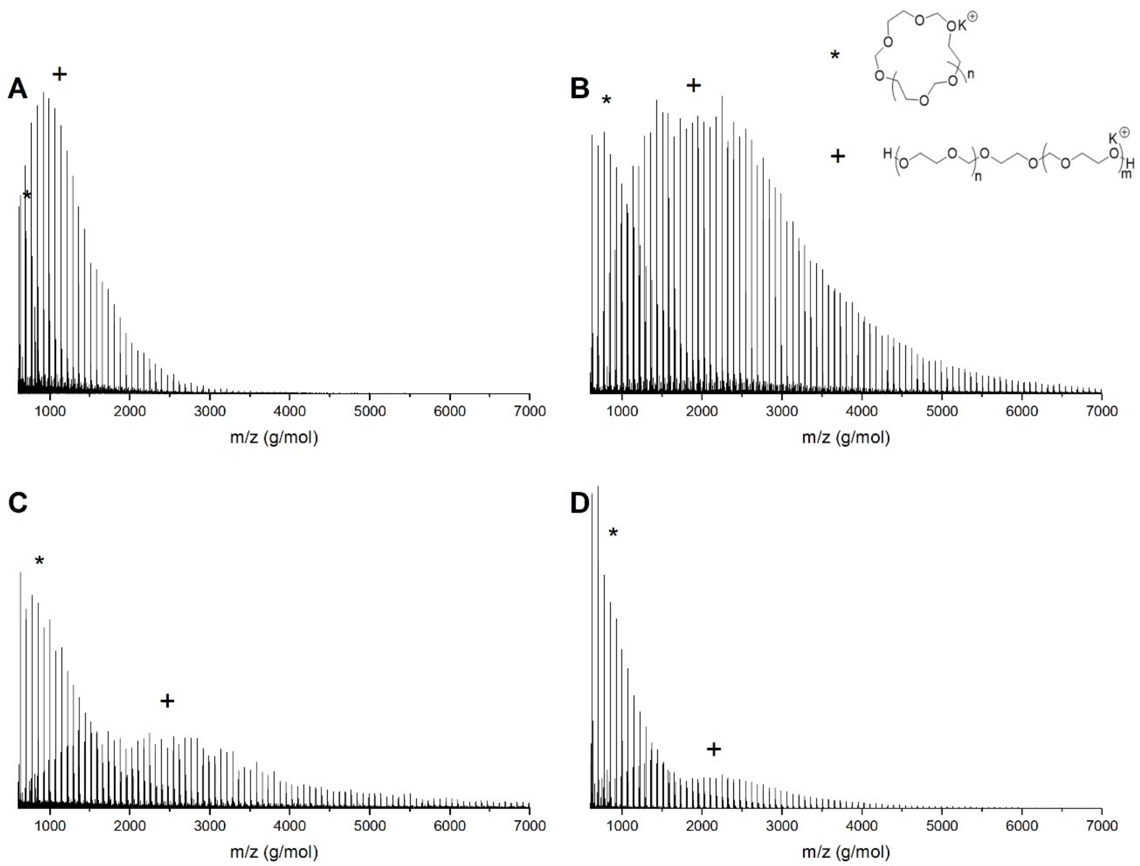

Figure S6 MALDI-ToF spectra of polymerization 2 (Table 1) before precipitation at (A) 5%, (B) 20%, (C) 52% and (D) 70% conversion.

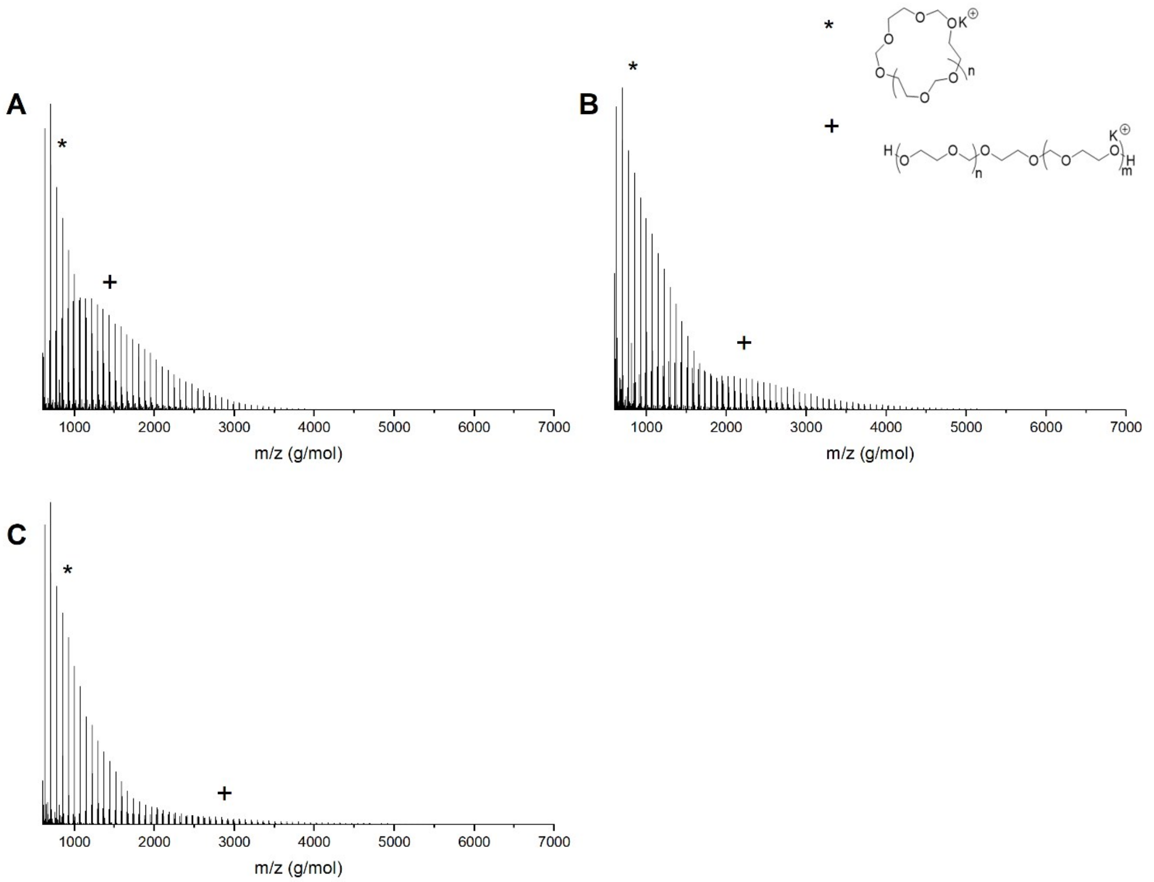

Figure S7 MALDI-ToF spectra of polymerization 3 (Table 1) before precipitation at (A) 7%, (B) 22% and (C) 70 % conversion.

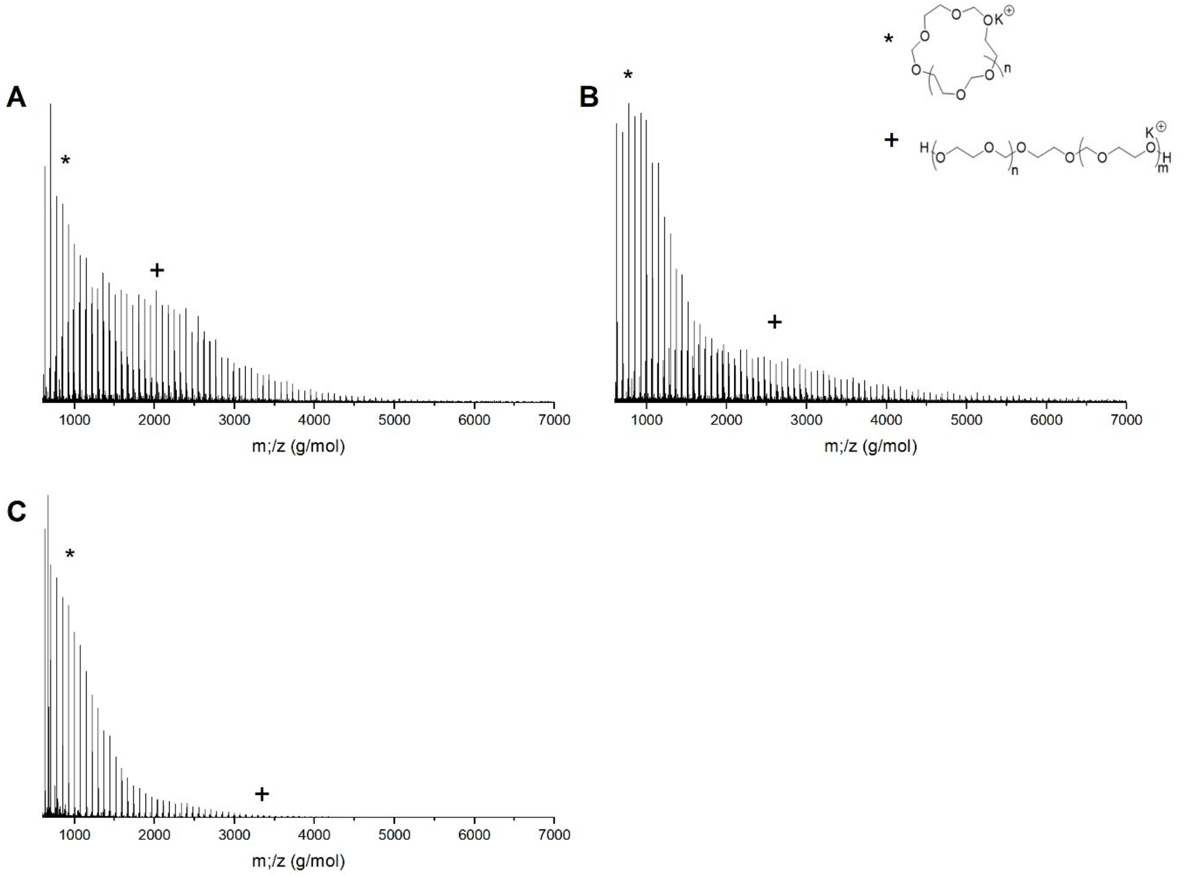

Figure S8 MALDI-ToF spectra of polymerization 4 (Table 1) before precipitation at (A) 9%, (B) 22% and (C) 59% conversion.

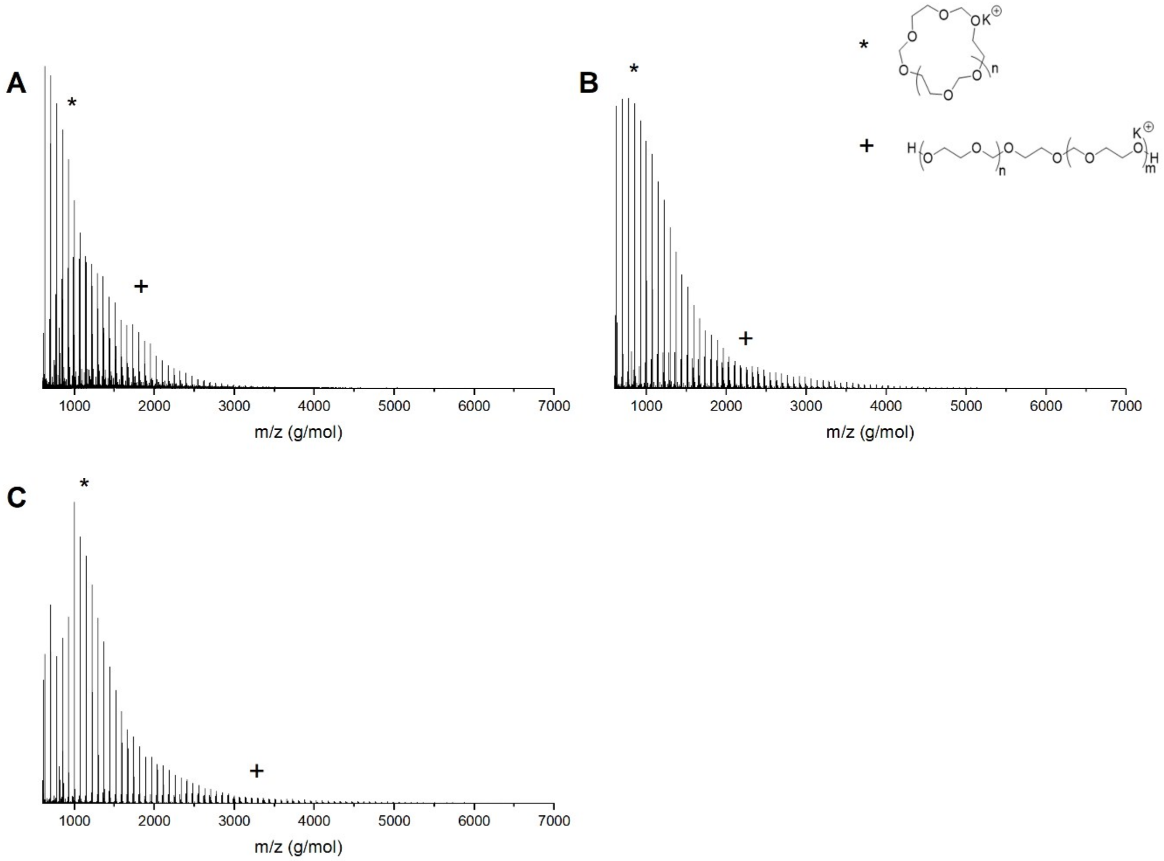

Figure S9 MALDI-ToF spectra of polymerization 5 (Table 1) before precipitation at (A) 4%, (B) 15% and (C) 48% conversion.

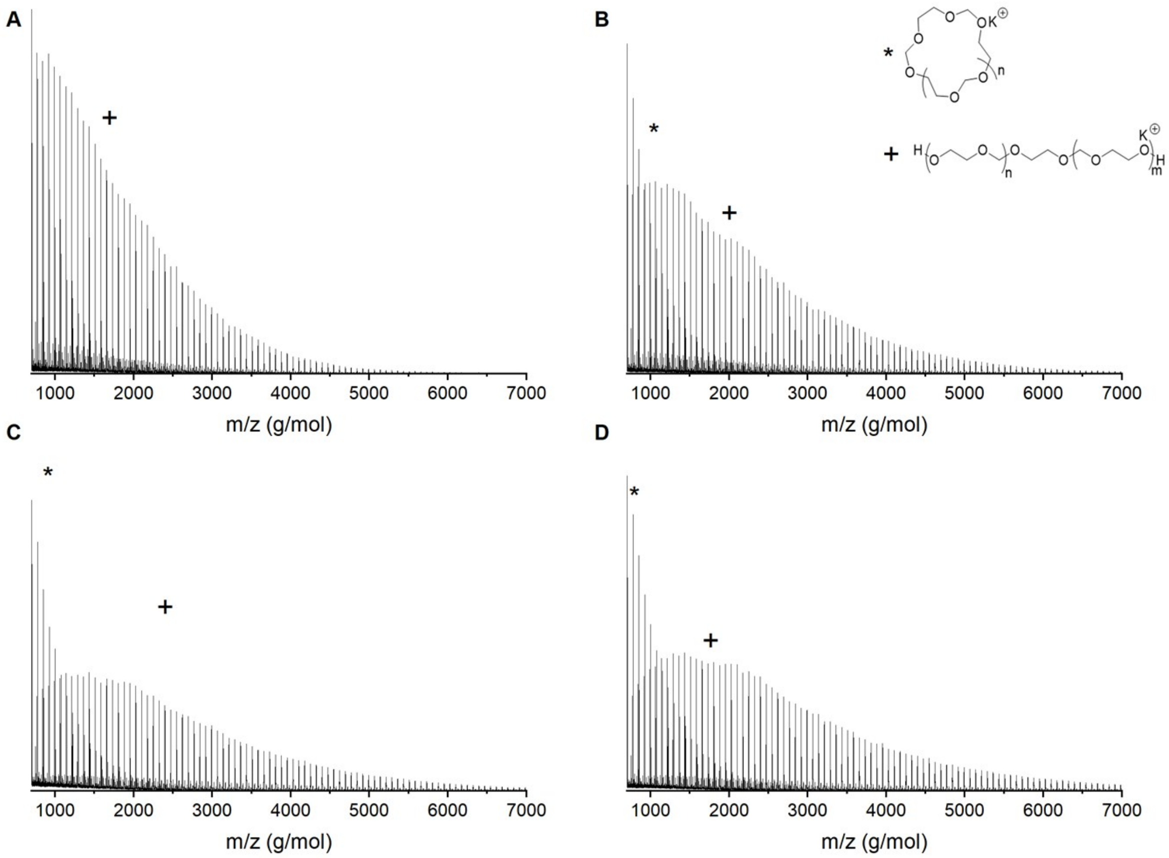

Figure S10 MALDI-ToF spectra of polymerization 11 (Table 1) before precipitation at (A) 15%, (B) 25%, (C) 40% and (D) 51% conversion.

MALDI-ToF MS of purified samples of polymerizations 1-5 measured in reflectron mode

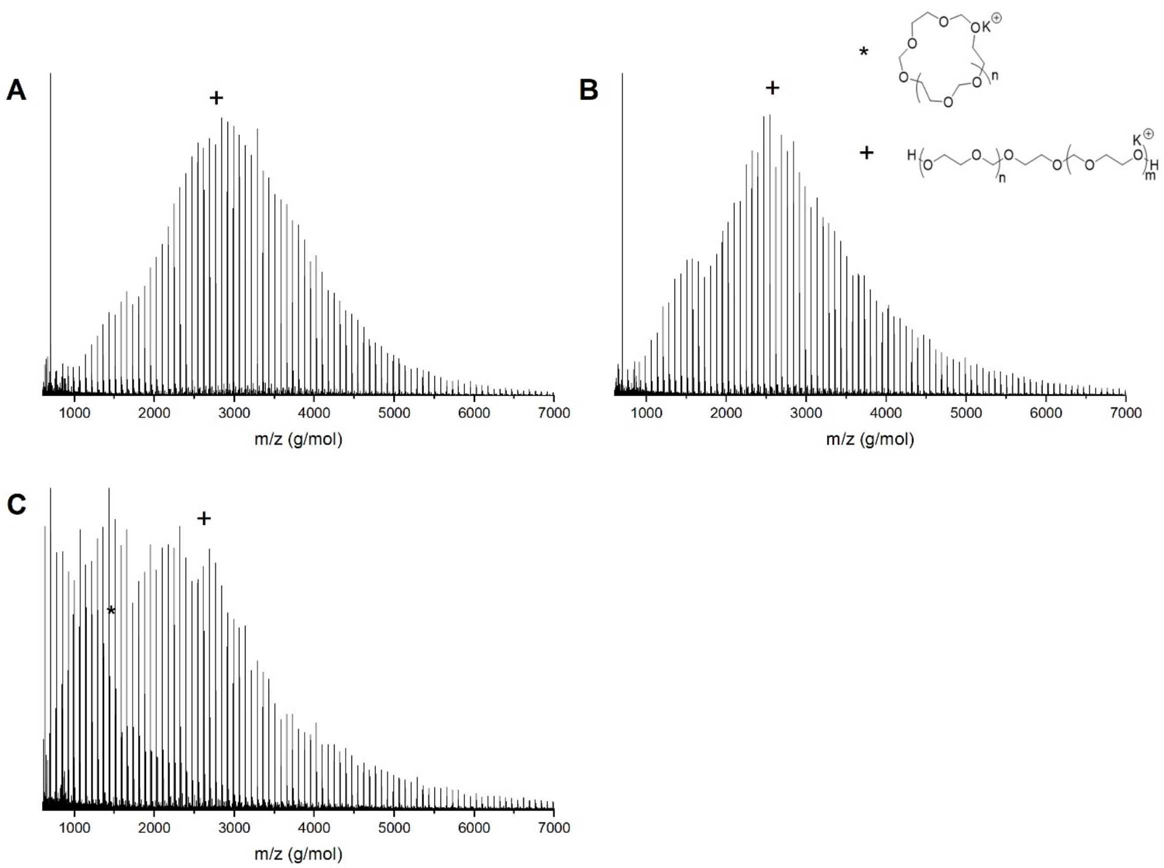

Figure S11. MALDI-ToF spectra of polymerization 1 (Table 1) after precipitation at (A) 25%, (B) 37% and (C) 65% conversion.

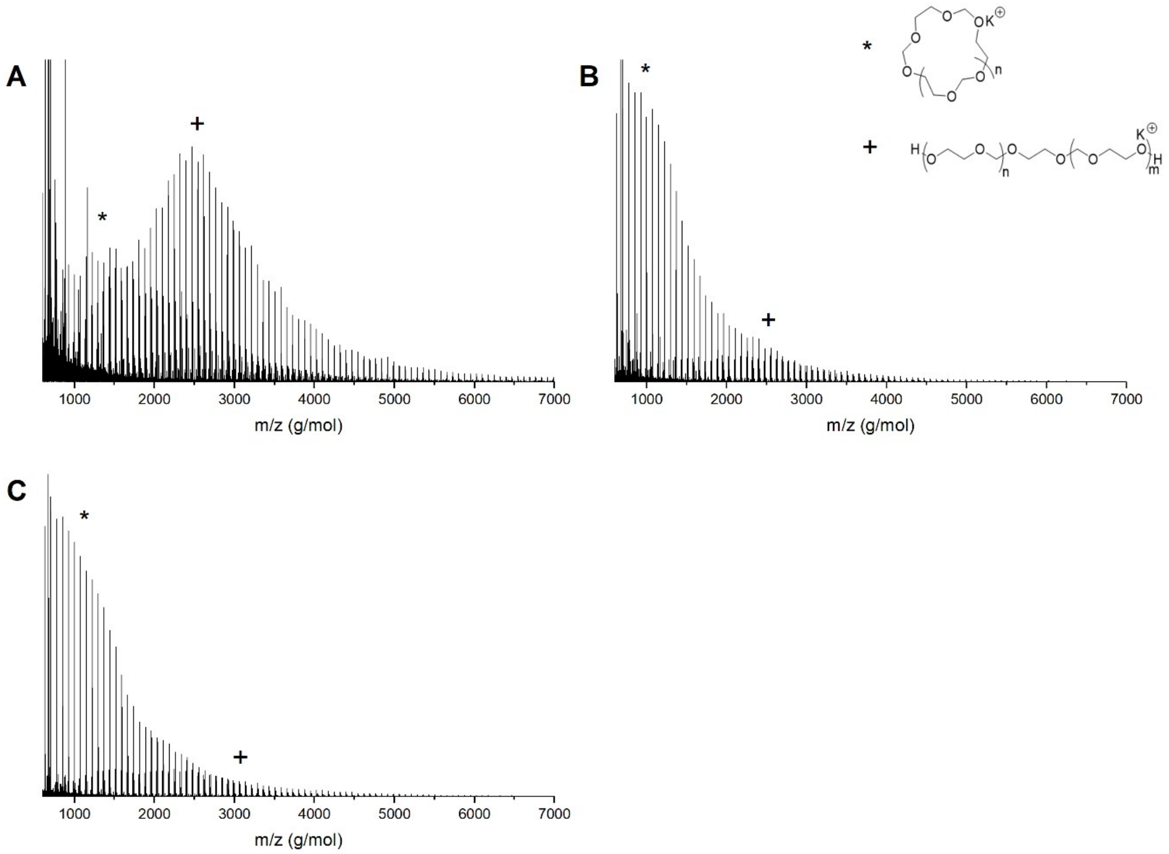

Figure S12. MALDI-ToF spectra of polymerization 2 (Table 1) after precipitation at (A) 26%, (B) 52% and (C) 70 % conversion.

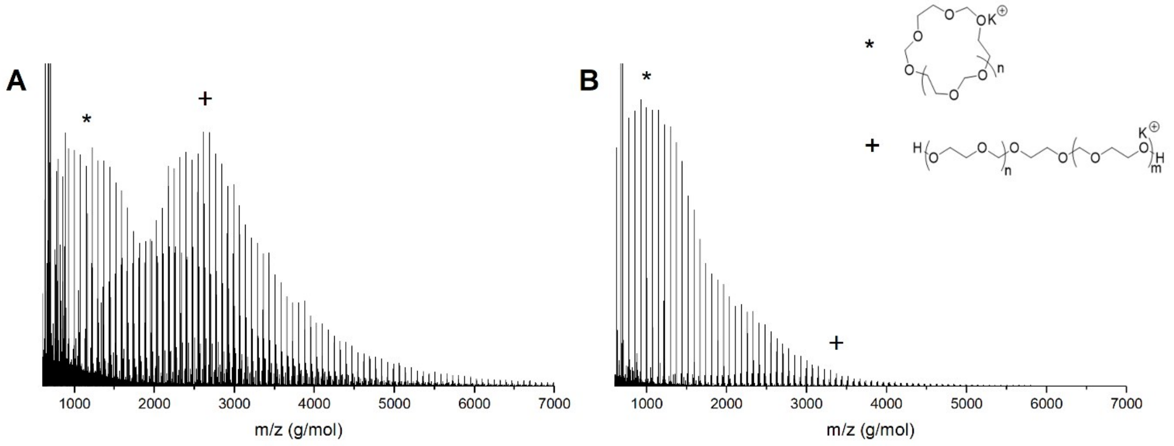

Figure S13. MALDI-ToF spectra of polymerization 3 (Table 1) after precipitation at (A) 30% and (B) 70% conversion.

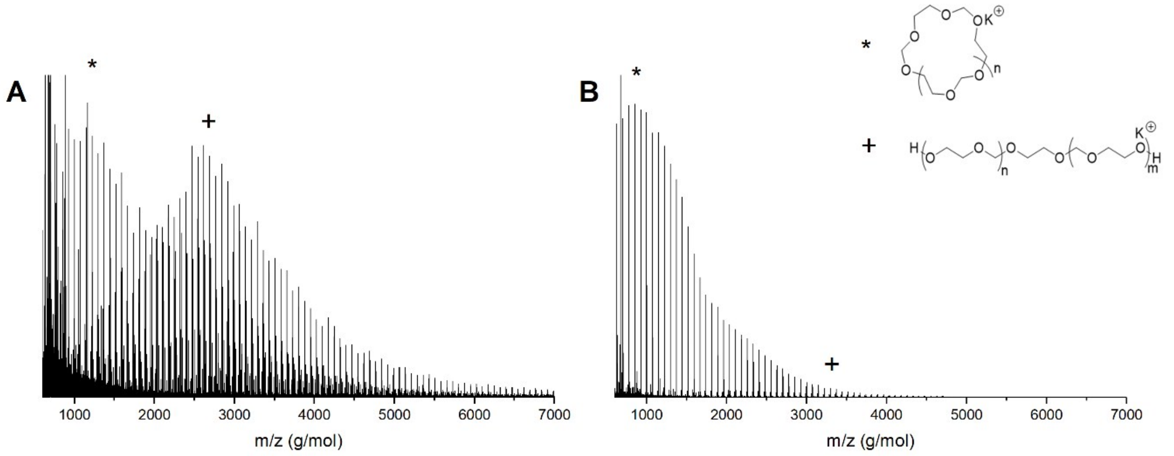

Figure S14. MALDI-ToF spectra of polymerization 4 (Table 1) after precipitation at (A) 22% and (B) 59% conversion.

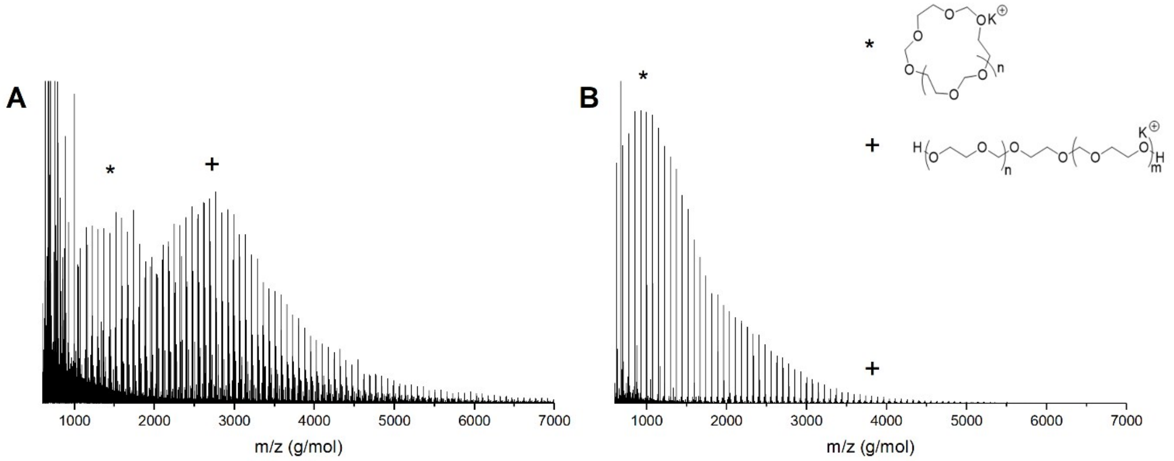

Figure S15. MALDI-ToF spectra of polymerization 5 (Table 1) after precipitation at (A) 22% and (B) 48% conversion.

^{31}P spectra of polymerization 11

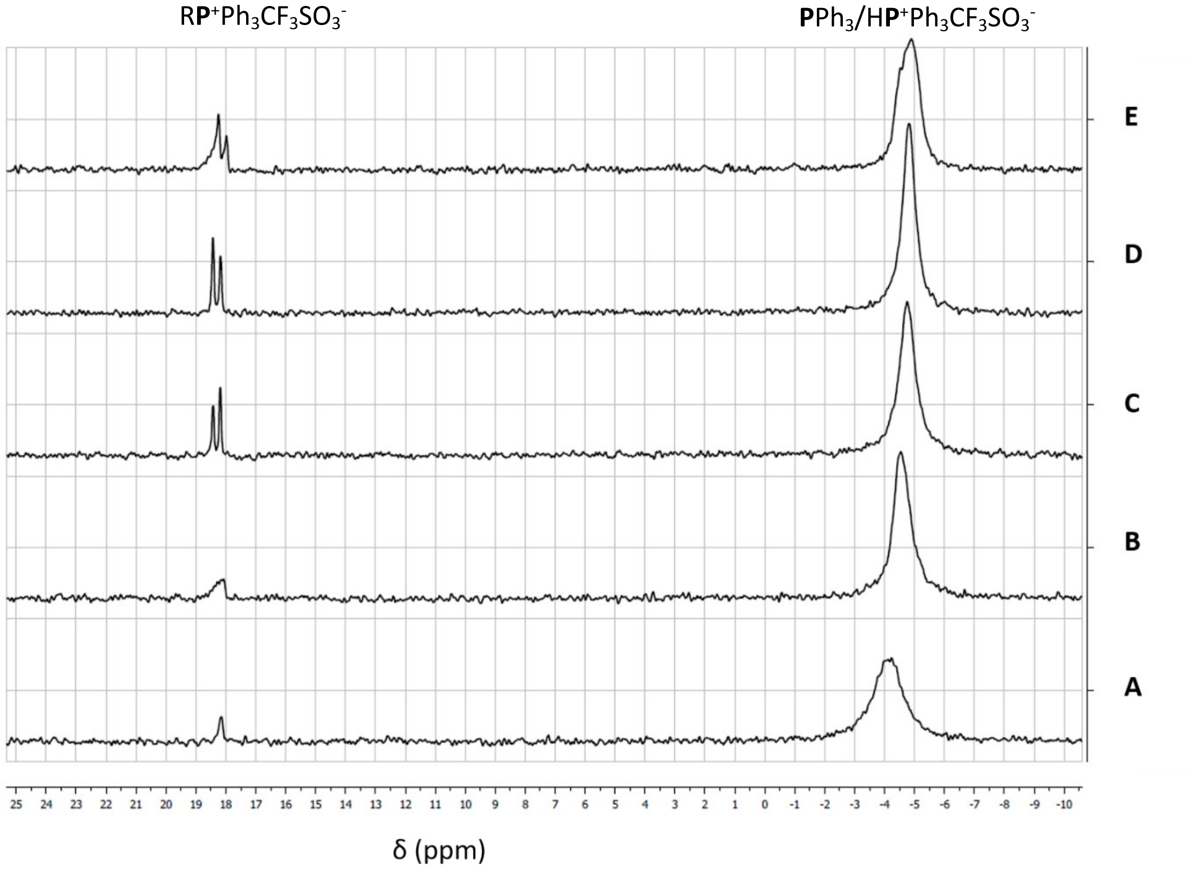

Figure S16. ^{31}P spectrum of polymerization 11 (Table 1) at (A) 0%, (B) 15%, (C) 25%, (D) 40% and (E) 51% conversion. R = polydioxolane.
